# Effect of Treatment with Mucoactive Drugs on COPD Exacerbations During 5 years of Follow-up in the Czech Republic: A Real-World Study

**DOI:** 10.1007/s00408-025-00813-7

**Published:** 2025-05-06

**Authors:** Jaromír Zatloukal, Clive Page, Kristián Brat, Michal Svoboda, Eva Voláková, Marek Plutinský, Michal Kopecký, Vladimír Koblížek

**Affiliations:** 1 Department of Respiratory Medicine, University Hospital Olomouc, Olomouc, Czech Republic; 2 Faculty of Medicine and Dentistry, Palacky University, Olomouc, Czech Republic; 3 Institute of Pharmaceutical Science, King’s College London, London, UK; 4 Department of Pulmonary Diseases and Tuberculosis, Masaryk University, Brno, Czech Republic; 5 Faculty of Medicine, Masaryk University, Brno, Czech Republic; 6 Institute of Biostatistics and Analyses Ltd, Brno, Czech Republic; 7 Institute of Biostatistics and Analyses, Faculty of Medicine, Masaryk University, Brno, Czech Republic; 8 Department of Pneumology, University Hospital, Hradec Kralove, Czech Republic; 9 Faculty of Medicine in Hradec Kralove, Charles University, Prague, Czech Republic

**Keywords:** Chronic obstructive pulmonary disease, Exacerbations, Mucoactive, Erdosteine, Cough

## Abstract

**Introduction:**

Studies indicate that chronic treatment with mucoactive drugs may reduce COPD exacerbation rates. This real-world, multicenter, prospective, observational study aimed to determine the effect of long-term mucoactive treatment on exacerbations in patients with COPD in the Czech Republic.

**Methods:**

452 adult patients on the Czech Multicenter Research Database of COPD with post-bronchodilator FEV_1_ ≤ 60% of predicted value received standard of care and were followed up for 5 years. For the first 24 months, 81 patients received regular thiol-based mucoactive drugs (77 erdosteine, 4 N-acetylcysteine) at the discretion of the treating physician and 371 patients had no mucoactive treatment (control group). Erdosteine was fully reimbursed, and NAC was partially reimbursed for COPD patients. The annual number/rate of COPD exacerbations over 5 years was monitored.

**Results:**

Patients receiving mucoactive treatment for 24 months had a significantly larger reduction from baseline in all exacerbations compared to the control group (− 0.61 vs − 0.18, *p* = 0.026; − 0.54 vs − 0.09, *p* = 0.007; − 0.55 vs 0.04, *p* = 0.005; − 0.67 vs 0.13, *p* = 0.002; − 0.53 vs 0.10, *p* = 0.019 in the first to fifth year, respectively). The reduction in moderate exacerbations was also significantly larger in those receiving mucoactive treatment versus no mucoactive treatment. The exacerbation rate was reduced to a greater extent in the subgroups with cough or with stage 3‒4 COPD who received mucoactive treatment but was independent of the use of inhaled corticosteroids (ICS).

**Conclusion:**

Mucoactive treatment for two years reduced the number of COPD exacerbations (all, moderate) over five years of follow-up. The reduction in exacerbations was more pronounced in patients with cough or with stage 3‒4 COPD but was independent of the use of ICS.

**Supplementary Information:**

The online version contains supplementary material available at 10.1007/s00408-025-00813-7.

## Introduction

Exacerbations of COPD negatively impact health status, rates of hospitalization and readmission, disease progression and mortality [1]. Exacerbations and persistent dyspnea are two key “treatable traits” of COPD in the Global Initiative for Chronic Obstructive Lung Disease (GOLD) report [1]. Therefore, treatment of patients with COPD aims to prevent or reduce exacerbations.

Mucus hypersecretion is a clinical feature of COPD and associated with symptoms of cough and expectoration [2, 3]. It can lead to airway obstruction, compromised mucociliary function, and bacterial colonization, resulting in repeated infections and exacerbations [4]. Mucoactive drugs are designed to alter the viscoelastic properties of mucus and promote secretion clearance. They can be classified based on their mechanism of action as expectorants, mucoregulators, mucolytics or mucokinetics [2]. Thiol-based drugs (erdosteine, N-acetylcysteine [NAC]) are considered as mucolytics because they decrease the viscosity and elasticity of bronchial secretions by reducing disulfide bonds in mucus proteins [5, 6]. They can also act as antioxidants, inhibit inflammation, and modulate human bronchial tone [5, 6]. Beyond that, thiol-based drugs reduce bacterial adhesion to the respiratory epithelial cell surface and inhibit biofilm formation, causing biofilm disruption and enhancing the efficacy of antibiotic therapy [5]. Therefore, we use the term “mucoactive drugs” or “mucoactive treatment” for thiol-based drugs.

Clinical studies have investigated the effect of mucoactive drugs on COPD exacerbations [7–11]. Notably, the RESTORE study (Reducing Exacerbations and Symptoms by Treatment with Oral Erdosteine in COPD), a 1-year randomized, placebo-controlled study of erdosteine added to usual COPD therapy, showed that patients with a history of moderate or severe exacerbations treated with erdosteine had a decreased exacerbation rate and shortened duration of events, especially when the patients had less severe COPD and more mild exacerbations [8, 10]. The current position of mucoactive drugs in treatment guidelines for COPD reflects the above studies [12–18]. However, there remains limited information on the real-world use of mucoactive treatment to reduce COPD exacerbations [1].

To better understand the role of mucoactive drugs in the treatment of patients with COPD, we have conducted a real-world study in a cohort of patients with COPD who were followed for 5 years. We compared COPD exacerbation rates in those treated with versus without mucoactive agents for the first 24 months to determine the effect of chronic mucoactive treatment on exacerbations in routine clinical practice settings.

## Methods

This real-world, multicenter, prospective, observational study compared the effect of regular mucoactive treatment for 24 months with no mucoactive treatment on the annual exacerbation rate over 5 years of follow-up in COPD patients.

### Study Design and Participants

We extracted data from the Czech Multicenter Research Database of COPD (CMRDC), a project registered at ClinicalTrials.gov (NCT01923051) and at the Czech Republic State Institute for Drug Control (identifier number 1301100001) [19]. The design of the research database, inclusion and exclusion criteria, ethical approval and other methodology details have been reported elsewhere [19]. Briefly, the CMRDC was a prospective, multicenter, observational database of patients with COPD (post-bronchodilator FEV_1_ ≤ 60% predicted), with patient follow-up every 6 months.

A total of 784 consecutive patients were recruited into the CMRDC between February 2013 and December 2016 from 14 centers providing respiratory care across the Czech Republic by their treating physicians. Inclusion criteria were age 18 years and older, a diagnosis of COPD, and a post-bronchodilator FEV_1_ ≤ 60% of predicted value. Patients without a confirmed diagnosis of COPD or patients in terminal stages of a malignancy or end-stage COPD (both with predicted survival < 3 months) or patients with exacerbation within 8 weeks prior to enrollment were excluded. There were no other exclusion criteria. All participants had access to full and complex medical care and were treated in the usual way according to the decision and practice of the treating physician. Patients were followed up after recruitment for five years or until death; the 5-year follow-up was completed in December 2021. Data collected included patient history, demographics, lung function tests, quality of life measures, symptoms, details of treatment, and assessment of exacerbations [19].

Disease exacerbations were identified by targeted inquiry and a search of hospital records. Information on the treatment of COPD exacerbations, hospitalizations (pulmonary and other) and their course was recorded. A moderate exacerbation of COPD was defined as a deterioration of COPD symptoms and the need for antibiotic treatment and/or systemic corticosteroids (oral or intravenous). A severe exacerbation of COPD was defined as the need for hospitalization or a visit to the emergency room. For the current analysis, the baseline exacerbation rate for each patient was the number of exacerbations in the 12 months prior to enrollment/registration on the database. The exacerbation rate was determined for each 12-month period of the 5-year follow-up.

All participants were treated according to the routine practice of their treating physicians who were free to prescribe whatever maintenance therapy they considered appropriate, which could include a long-acting muscarinic antagonist (LAMA), a long-acting β_2_-agonist (LABA), an ICS, or any other treatment prescribed for COPD, alone or in combination. Mucoactive treatment was also assigned to the patient only according to the physician's decision and practice. It is possible that the physicians followed the Czech national guideline based on clinical phenotypes, which was already valid at the time and which recommended phenotype-specific treatment for each clinical phenotype. In the Czech Republic, the available mucoactive drugs are erdosteine (300 mg twice daily) or NAC (600 mg once daily). Erdosteine is fully reimbursed, and NAC is partially reimbursed for COPD patients.

At 24 months following database enrollment, each patient was assigned to one of two cohorts or excluded from the analysis. The treatment cohort included all patients who were treated regularly with a mucoactive drug for the first 24 months and the control cohort included all patients not treated with a mucoactive drug. Patients treated with a mucoactive drug irregularly or for only part of the first 24-month period were excluded from further analysis. During the follow-up period (years 3 to 5), patients from both the treated and control cohorts may or may not have received mucoactive therapy.

### Study Outcomes

The primary objective of this study was to determine the frequency of all exacerbations per year over a 5-year period in patients treated for the first 24 months with mucoactive therapy versus patients without mucoactive therapy, on top of standard of care, and to determine the change from baseline in exacerbation rate for each 12-month period.

Secondary objectives were to determine the change from baseline in exacerbations in patients having moderate and severe exacerbations, and in subgroups of patients with chronic cough at baseline (cough lasting 8 weeks or longer), with severe or very severe COPD at baseline (GOLD stages 3 or 4), in patients with and without concurrent ICS use during mucoactive treatment, and in patients with treatable traits corresponding to certain phenotypes (bronchitic, frequent exacerbators, bronchiectasis-COPD overlap).

### Statistical Analysis

Baseline characteristics are described overall and by patient cohort (treatment, control) using mean (SD) for continuous measures and numbers (%) for categorical measures. Percentages were calculated from known data. Pearson’s chi-squared tests or Fisher’s exact tests were used to analyze differences between the treatment and control groups for categorical variables, and two-sample t-tests or Mann–Whitney U tests for continuous variables, depending on normality of data. Linear mixed models were used to analyze an influence of the treatment on number of exacerbations. This influence was also adjusted by FEV_1_ (% predicted), cough, number of exacerbations at the baseline visit and ICS treatment. Analysis was performed in software R, version 4.2.0. All hypotheses were tested on 5% level of significance.

## Results

**Fig. 1:**
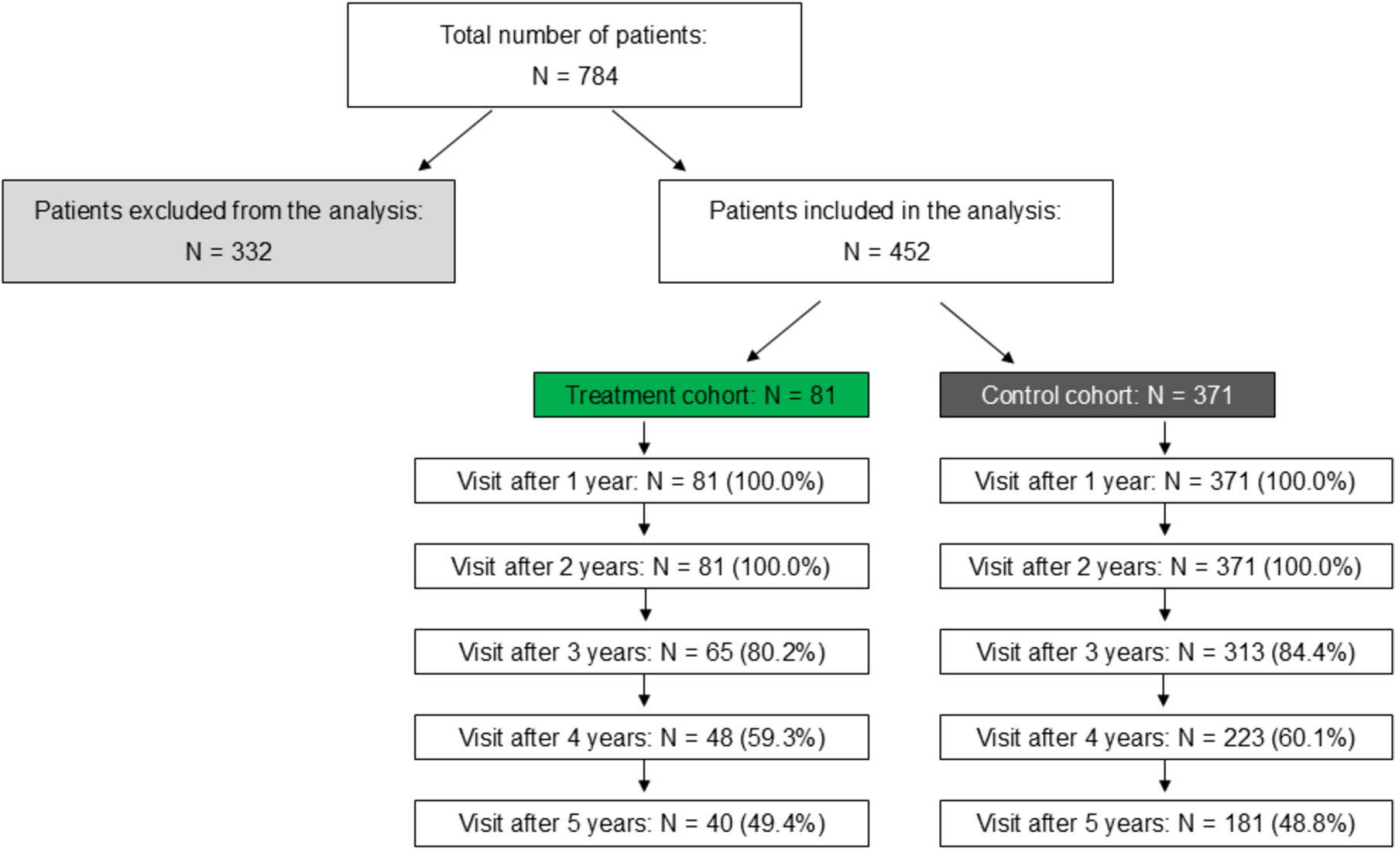
Study design

Of 784 patients registered in the CMRDC, 81 were treated regularly with mucoactive drugs for the first 24 months (treatment cohort), 371 did not receive mucoactive drugs (control cohort), and the remaining 332 patients were excluded from the analysis (Fig. 1). Baseline characteristics of included and excluded patients is shown in Supplementary Table S1. Patients who used mucoactive therapy irregularly during the first 24 months and were excluded from the analysis had worse CAT and FEV_1_ and had more exacerbations. Of the patients included in the analysis, 77 patients in the treatment cohort were prescribed erdosteine and 4 patients were prescribed NAC. None of the patients used both mucoactive drugs.

Baseline characteristics are detailed in Table 1. The treatment and control cohorts were comparable for sex, age, smoking status, BMI, and level of dyspnea. The study population had a mean age of 65.7 years, with 73.2% males, a mean BMI of 28.4 kg/m^2^, mean dyspnea mMRC index of 2.2, and 88.5% patients were active or former smokers. There was a slightly higher frequency of severe (GOLD stage 3) or very severe (GOLD stage 4) COPD in the treatment vs. control cohort (56.2% vs. 46% and 13.7% vs. 9.5%, respectively; *p* = 0.071). At baseline, patients in the treatment cohort had a worse COPD assessment test (CAT) score (18.9 vs. 14.0; *p* < 0.001), worse FEV_1_ (43.5% vs. 47.4% predicted; *p* = 0.011), and a higher prevalence of chronic cough (85.2% vs. 67.9%; *p* = 0.002) and expectoration (79.0% vs. 50.9%; *p* < 0.001) than patients in the control cohort. At baseline, 51.8% of all patients had no exacerbations in the previous 12 months, but the proportion of patients with one or more exacerbations was higher in the treatment cohort vs. control cohort (*p* < 0.001). The mean exacerbation rate at baseline was higher in the treatment vs. control cohort for all exacerbations (1.6 vs. 0.9; *p* < 0.001), moderate exacerbations (1.1 vs. 0.6; *p* < 0.001), and severe exacerbations (0.6 vs. 0.2; *p* < 0.001). Patients in the treatment cohort were more frequent users of LAMA, LABA, ICS, and roflumilast, and 74.1% had been treated with a mucoactive (erdosteine or NAC) before entry into the study.

**Table 1:** Baseline demographic and clinical characteristics

		All patients (*N* = 452)	Treatment cohort (*N* = 81)	Control cohort (*N* = 371)	*P*-value
Male	N (%)	331 (73.2)	57 (70.4)	274 (73.9)	0.521
Age (years)	Mean (SD)	65.7 (9.5)	67.0 (7.8)	65.5 (9.8)	0.343
Smoking status	Current smoker, n (%)	87 (19.2)	15 (18.5)	72 (19.4)	0.957
	Ex-smoker, n (%)	313 (69.2)	56 (69.1)	257 (69.3)	
	Non-smoker, n (%)	52 (11.5)	10 (12.3)	42 (11.3)	
BMI (kg/m^2^)	Mean (SD)	28.4 (6.2)	28.1 (5.2)	28.5 (6.3)	0.947
Dyspnea (mMRC)	Mean (SD)	2.2 (1.0)	2.3 (1.1)	2.2 (1.0)	0.229
CAT score	Mean (SD)	14.9 (7.5)	18.9 (7.0)	14.0 (7.3)	< 0.001*
Chronic cough	N (%)	321 (71.0)	69 (85.2)	252 (67.9)	0.002*
Expectoration	N (%)	253 (56.0)	64 (79.0)	189 (50.9)	< 0.001*
FEV_1_ (% predicted)	Mean (SD)	46.7 (11.6)	43.5 (11.9)	47.4 (11.4)	0.011*
GOLD stage^a^, n (%)	2	172 (42.0)	22 (30.1)	150 (44.5)	0.071
	3	196 (47.8)	41 (56.2)	155 (46.0)	
	4	42 (10.2)	10 (13.7)	32 (9.5)	
	Unknown	42	8	34	
Clinical phenotype	Bronchitic	253 (56.0)	64 (79.0)	189 (50.9)	< 0.001*
	Emphysematic	165 (73.7)	34 (73.9)	131 (73.6)	0.965
	BCO	61 (27.4)	18 (39.1)	43 (24.3)	0.044*
	ACO	17 (4.7)	0 (0.0)	17 (5.6)	0.086
	Frequent exacerbator	113 (25.0)	39 (48.1)	74 (19.9)	< 0.001*
	Pulmonary cachexia	42 (9.3)	7 (8.6)	35 (9.4)	0.824
Exacerbations in previous 12 months, mean (SD)	All	1.0 (1.5)	1.6 (1.4)	0.9 (1.5)	< 0.001*
	Moderate	0.7 (1.3)	1.1 (1.1)	0.6 (1.3)	< 0.001*
	Severe	0.3 (0.7)	0.6 (1.0)	0.2 (0.6)	< 0.001*
Frequency of all exacerbations in previous 12 months, n (%)	0	234 (51.8)	23 (28.4)	211 (56.9)	< 0.001*
	1	105 (23.2)	19 (23.5)	86 (23.2)	
	2	54 (11.9)	15 (18.5)	39 (10.5)	
	3	29 (6.4)	14 (17.3)	15 (4.0)	
	> 3	30 (6.6)	10 (12.3)	20 (5.4)	
Treatment at baseline, n (%)	Containing ICS	241 (53.3)	51 (63.0)	190 (51.2)	0.055
	Containing LABA	386 (85.4)	76 (93.8)	310 (83.6)	0.018*
	Containing LAMA	324 (71.7)	71 (87.7)	253 (68.2)	< 0.001*
	LAMA + LABA	171 (37.8)	44 (54.3)	127 (34.2)	< 0.001*
	LAMA + LABA + ICS	183 (40.5)	46 (56.8)	137 (36.9)	< 0.001*
	Erdosteine	61 (13.5)	57 (70.4)	4 (1.1)	< 0.001*
	N-acetylcysteine	3 (0.7)	3 (3.7)	0 (0.0)	0.006*
	Theophylline	208 (46.0)	35 (43.2)	173 (46.6)	0.576
	Roflumilast	45 (10.0)	21 (25.9)	24 (6.5)	< 0.001*

*ACO* Asthma-COPD overlap, *BCO* bronchiectasis with COPD, *BMI* Body Mass Index, *CAT* COPD Assessment Test (score range 0–40), *FEV*_*1*_ Forced Expiratory Volume in one second, *ICS* Inhaled corticosteroid, *LABA* long-acting muscarinic antagonist, *LAMA* long-acting beta_2_-agonist, *mMRC* modified Medical Research Council dyspnea scale

^a^GOLD stage 2, 50% ≤ FEV_1_ < 80% predicted; GOLD stage 3, 30% ≤ FEV_1_ < 50% predicted; GOLD stage 4, FEV_1_ < 30% predicted

^*^Statistically significant difference between treatment and control cohorts

A multivariate adjustment was performed to control for baseline disparities of treatment and control cohort. The results are presented in the Supplementary Table S2 and they confirm statistical significance of differences in exacerbation reduction. Annual change of number of exacerbations was 0.06 in the control cohort. Annual change of number of exacerbations was − 0.13 in the treatment cohort. This difference (− 0.19) is statistically significant (*p* < 0.001). Similar results were achieved with adjustment by confounding factors FEV_1_, cough, baseline number of exacerbations or ICS use during first 24 months (Supplementary Table S2b and S2c). Furthermore, multivariate models were performed as sensitivity analysis to prove a consistency of the results. Treatment was adjusted by FEV_1_, cough, and baseline number of exacerbations. Three analyses were performed based on the type of exacerbations. Linear model with mixed effects showed similar results in prediction of number of all and moderate exacerbations after these adjustments (*p* < 0.001 and *p* = 0.001, respectively). Analysis of severe exacerbations also shows similar results after adjustment, but due to the lower number of severe exacerbations, the differences do not reach statistical significance. Additional details on the sensitivity testing are also provided in Supplementary Table S3.

Patients in the treatment cohort were more likely to have a bronchitic phenotype, bronchiectasis-COPD overlap phenotype, or frequent exacerbator phenotype than patients in the control cohort. The baseline characteristics of the subgroups of patients with these phenotypes are shown in Supplementary Table S8. The percentage of patients who discontinued due to loss to follow-up or death did not differ between the cohorts; 49.4% of patients in treatment cohort and 48.8% patients in control cohort remained in the study at Year 5 (Fig. 1). Baseline characteristics of patients in treatment and control cohorts who dropped out during the study are shown in Supplementary Table S4. Causes of death of patients in the treatment and control cohorts who died during the study are shown in Supplementary Table S5. The percentage of patients with mucoactive treatment during the treatment period and follow-up are shown in Supplementary Table S6.

**Fig. 2:**
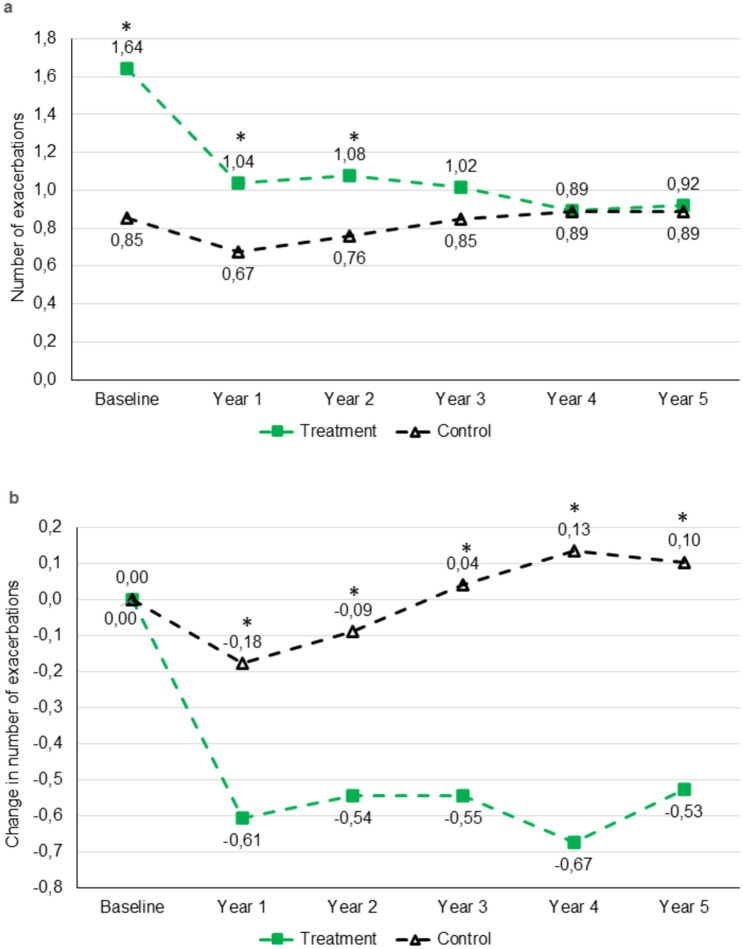
All exacerbations over 5 years in COPD patients treated with mucoactive drugs for 24 months (treatment) vs. no mucoactive treatment (control). **a** mean number of exacerbations. **b** mean change from baseline in number of exacerbations

The mean number of all exacerbations per year and the change from baseline over five years are shown in Table 2 and Fig. 2. Patients in the treatment cohort had a significantly higher mean exacerbation rate at baseline, but they also had a significantly larger reduction from baseline in exacerbation rate during all five years compared to the control cohort.

**Table 2:** All exacerbations: number of exacerbations per year and change from baseline in the treatment and control cohorts

	Number of exacerbations					Change from baseline				
	*n*	Treatment cohort	*n*	Control cohort	*P* value	*n*	Treatment cohort	*n*	Control cohort	*P* value
Baseline	81	1.64 (1.43)	371	0.85 (1.53)	< 0.001*	–	–	–	–	–
Year 1	81	1.04 (1.32)	368	0.67 (1.10)	0.022*	81	− 0.61 (1.61)	368	− 0.18 (1.47)	0.026*
Year 2	79	1.08 (1.35)	365	0.76 (1.37)	0.015*	79	− 0.54 (1.48)	365	− 0.09 (1.53)	0.007*
Year 3	66	1.02 (1.23)	307	0.85 (1.44)	0.074	66	− 0.55 (1.39)	307	0.04 (1.59)	0.005*
Year 4	46	0.89 (1.20)	216	0.89 (1.28)	0.916	46	− 0.67 (1.66)	216	0.13 (1.51)	0.002*
Year 5	38	0.92 (1.15)	174	0.89 (1.49)	0.375	38	− 0.53 (1.31)	174	0.10 (2.01)	0.019*

Data presented as mean (SD)

Only patients who had no missing data on exacerbations are included in the table

^*^Statistically significant difference between treatment and control cohorts

**Fig. 3:**
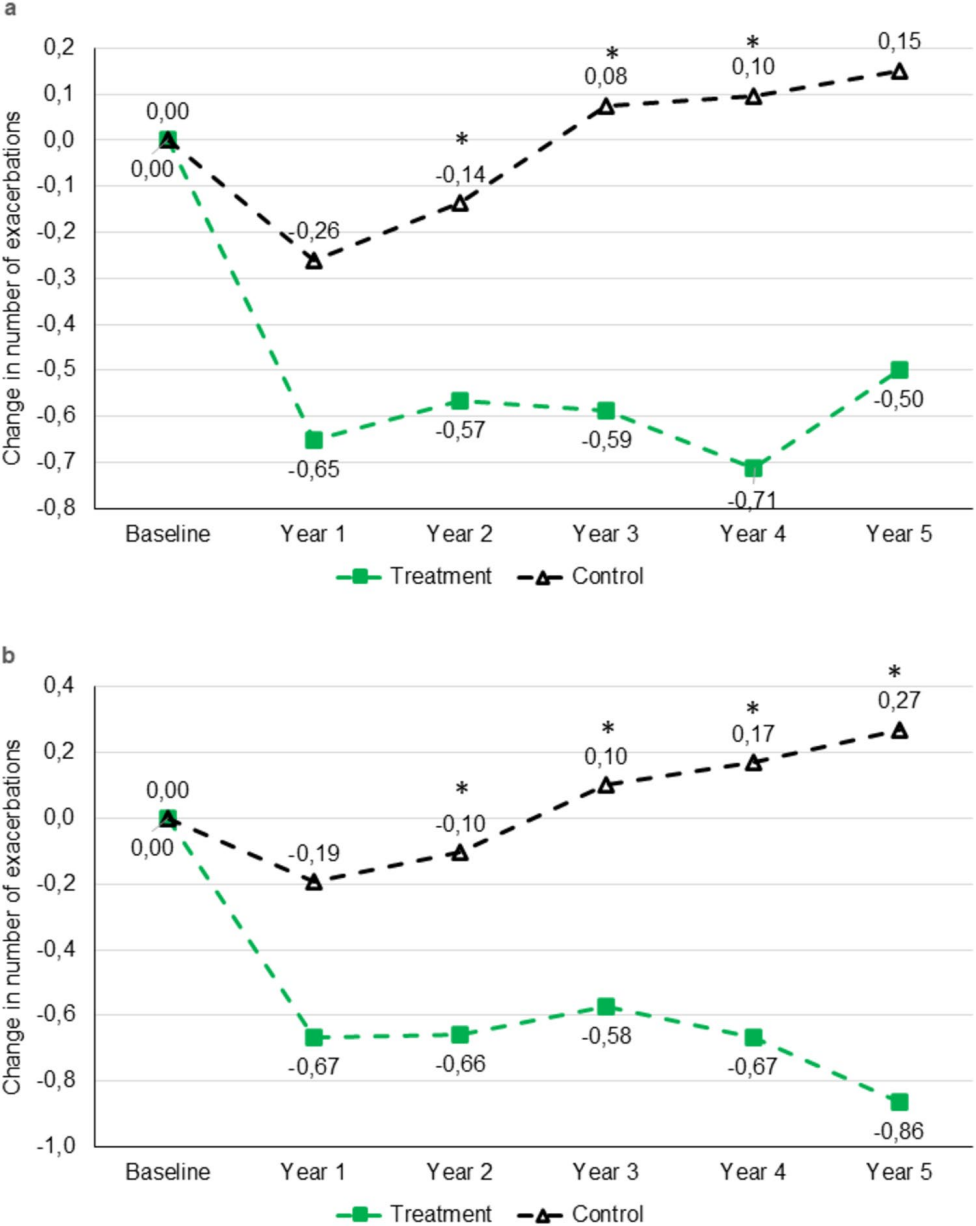
Mean change from baseline in number of all exacerbations over 5 years in subgroups of COPD patients treated with mucoactive drugs for 24 months (treatment) vs. no mucoactive treatment (control) with **a** cough at baseline or **b** severe/very severe COPD (GOLD stages 3‒4) at baseline

For the subgroups of patients with chronic cough at baseline or GOLD stages 3‒4 at baseline, the treatment cohort had a larger reduction from baseline of all exacerbations than the patients in the control cohort; the difference between groups was significant in three of the five years of follow-up in patients with cough at baseline and in four of the five years of follow-up in patients with GOLD stages 3‒4 at baseline (Table 3, Fig. 3).

**Table 3:** Number of all exacerbations and change from baseline in treatment and control cohorts in subgroups of patients with cough and patients with GOLD stage 3 or 4 at baseline

	Cough at baseline					GOLD Stage 3 or 4 at baseline				
	n	Treatment cohort	n	Control cohort	P value	n	Treatment cohort	*n*	Control cohort	*P* value
Baseline	69	1.74 (1.47)	252	0.98 (1.69)	< 0.001*	51	1.80 (1.51)	187	0.99 (1.73)	< 0.001*
Year 1	69	1.09 (1.37)	250	0.72 (1.15)	0.056	51	1.14 (1.30)	186	0.80 (1.17)	0.091
Year 2	67	1.15 (1.42)	249	0.85 (1.53)	0.041*	50	1.12 (1.26)	184	0.90 (1.54)	0.115
Year 3	56	1.09 (1.27)	210	1.01 (1.55)	0.250	40	1.23 (1.39)	155	1.04 (1.63)	0.192
Year 4	42	0.93 (1.22)	145	1.03 (1.38)	0.759	30	1.10 (1.27)	106	1.02 (1.40)	0.540
Year 5	34	1.03 (1.17)	112	1.05 (1.70)	0.405	22	0.96 (1.25)	89	1.12 (1.74)	0.981
Change from baseline										
Year 1	69	− 0.65 (1.69)	250	− 0.26 (1.59)	0.070	51	− 0.67 (1.68)	186	− 0.19 (1.51)	0.083
Year 2	67	− 0.57 (1.54)	249	− 0.14 (1.62)	0.028*	50	− 0.66 (1.45)	184	− 0.10 (1.55)	0.028*
Year 3	56	− 0.59 (1.41)	210	0.08 (1.70)	0.008*	40	− 0.58 (1.45)	155	0.10 (1.62)	0.027*
Year 4	42	− 0.71 (1.73)	145	0.10 (1.64)	0.007*	30	− 0.67 (1.71)	106	0.17 (1.62)	0.023*
Year 5	34	− 0.50 (1.35)	112	0.15 (2.26)	0.052	22	− 0.86 (1.39)	89	0.27 (2.15)	0.007*

Data presented as mean (SD)

Only patients who had no missing data on exacerbations are included in the table

^*^Statistically significant difference between treatment and control cohorts

**Fig. 4:**
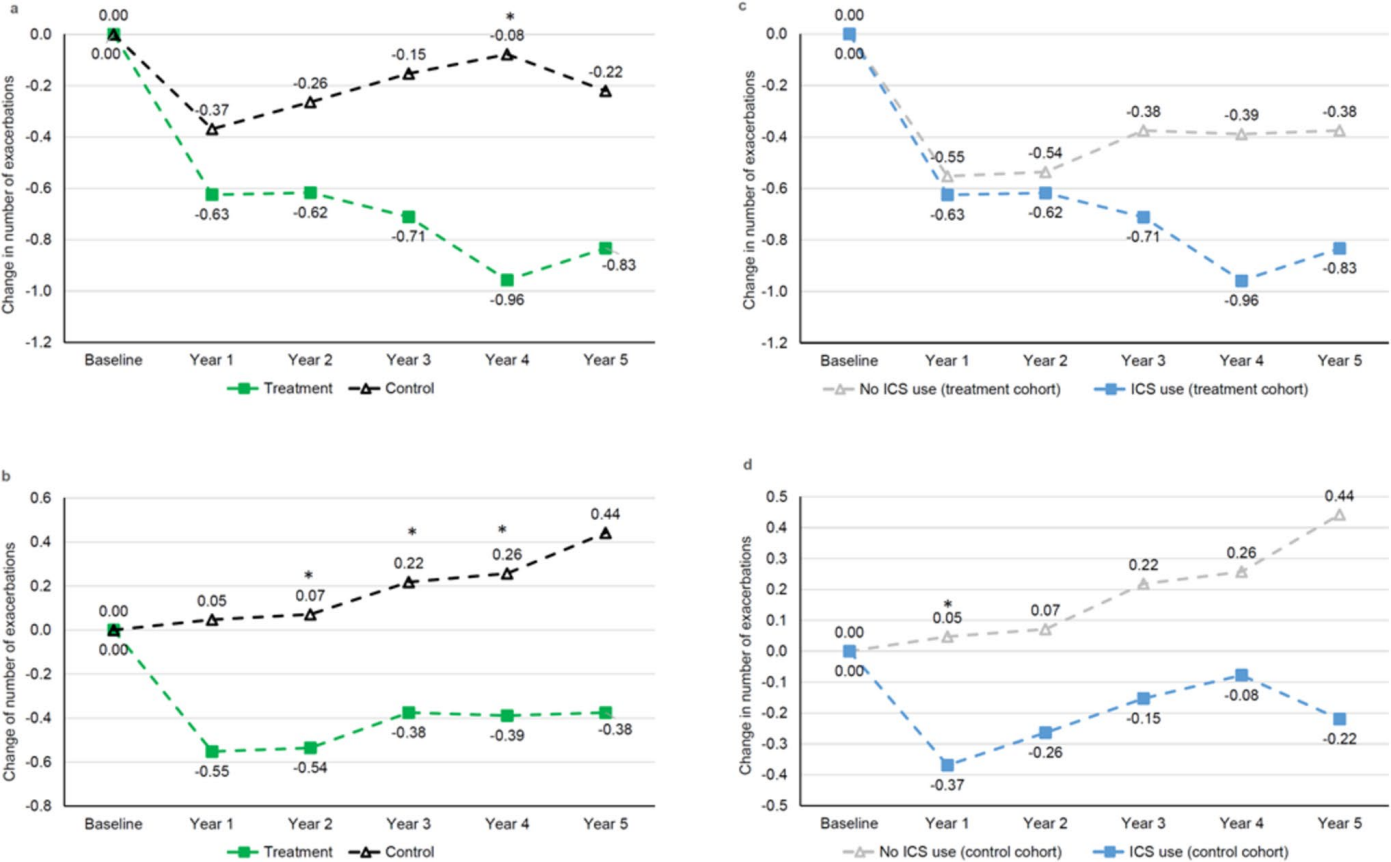
Mean change from baseline in number of all exacerbations over 5 years in **a** patients using ICS during first 24 months (treatment cohort vs. control cohort), **b** patients not using ICS during first 24 months (treatment cohort vs. control cohort), **c** ICS use vs. no ICS use during first 24 months (treatment cohort), **d** ICS use vs. no ICS use during first 24 months (control cohort). ICS use includes use of fixed ICS + LABA

Baseline characteristics of patients in the treatment cohort and in the control cohort treated concurrently with an ICS during the first 24 months or without concurrent ICS treatment during the first 24 months are shown in Supplementary Table S9. Patients in the mucoactive treatment cohort treated concurrently with an ICS during the first 2 years had a larger reduction from baseline in all exacerbations than patients in the control cohort using ICS; the difference between groups was significant in year 4 of follow-up (Table 4, Fig. 4a). Among patients without concurrent ICS use during the treatment period, the reduction in all exacerbations was larger in the treatment cohort vs. control cohort, which was significant in three of the five years of follow-up (Table 4, Fig. 4b). Comparisons between patients in the mucoactive treatment cohort treated with vs. without ICS during the first 24 months showed a non-significantly larger reduction from baseline in the mean number of exacerbations during this period in patients with concurrent ICS treatment (Supplementary Table S10, Fig. 4c). Among patients in the control cohort, the change from baseline in mean exacerbation rate was non-significantly greater in the subgroup with concurrent ICS use compared to the subgroup without ICS, except for a significantly larger reduction in exacerbations with the ICS users in year 1 (Supplementary Table S10, Fig. 4d).

**Table 4:** Number of all exacerbations and change from baseline in treatment and control cohorts in subgroups of patients with ICS use during for24 months and patients with no ICS use for 24 months

	ICS use during first 24 months					No ICS use during first 24 months				
	*n*	Treatment cohort	*n*	Control cohort	*P* value	*n*	Treatment cohort	*n*	Control cohort	*P* value
Baseline	48	1.94 (1.45)	180	1.20 (1.94)	< 0.001*	29	1.24 (1.38)	171	0.50 (0.86)	0.002*
Year 1	48	1.31 (1.39)	179	0.84 (1.23)	0.025*	29	0.69 (1.17)	170	0.55 (0.96)	0.65
Year 2	47	1.32 (1.48)	178	0.93 (1.48)	0.045*	28	0.64 (0.99)	169	0.58 (1.26)	0.489
Year 3	38	1.24 (1.38)	150	0.97 (1.39)	0.182	24	0.67 (0.96)	142	0.71 (1.48)	0.508
Year 4	24	1.00 (1.25)	103	1.04 (1.36)	0.969	18	0.78 (1.11)	105	0.69 (1.11)	0.764
Year 5	18	1.00 (1.03)	91	0.91 (1.27)	0.475	16	0.75 (1.29)	77	0.84 (1.75)	0.796
Change from baseline										
Year 1	48	− 0.63 (1.75)	179	− 0.37 (1.78)	0.326	29	− 0.55 (1.48)	170	0.05 (1.06)	0.053
Year 2	47	− 0.62 (1.65)	178	− 0.26 (1.68)	0.102	28	− 0.54 (1.20)	169	0.07 (1.35)	0.028*
Year 3	38	− 0.71 (1.63)	150	− 0.15 (1.71)	0.065	24	− 0.38 (0.97)	142	0.22 (1.48)	0.032*
Year 4	24	− 0.96 (2.01)	103	− 0.08 (1.70)	0.044*	18	− 0.39 (1.04)	105	0.26 (1.25)	0.033*
Year 5	18	− 0.83 (1.65)	91	− 0.22 (2.07)	0.081	16	− 0.38 (0.89)	77	0.44 (1.94)	0.074

Data presented as mean (SD)

Only patients who had no missing data on exacerbations are included in the table

^*^Statistically significant difference between treatment and control cohorts

**Fig. 5:**
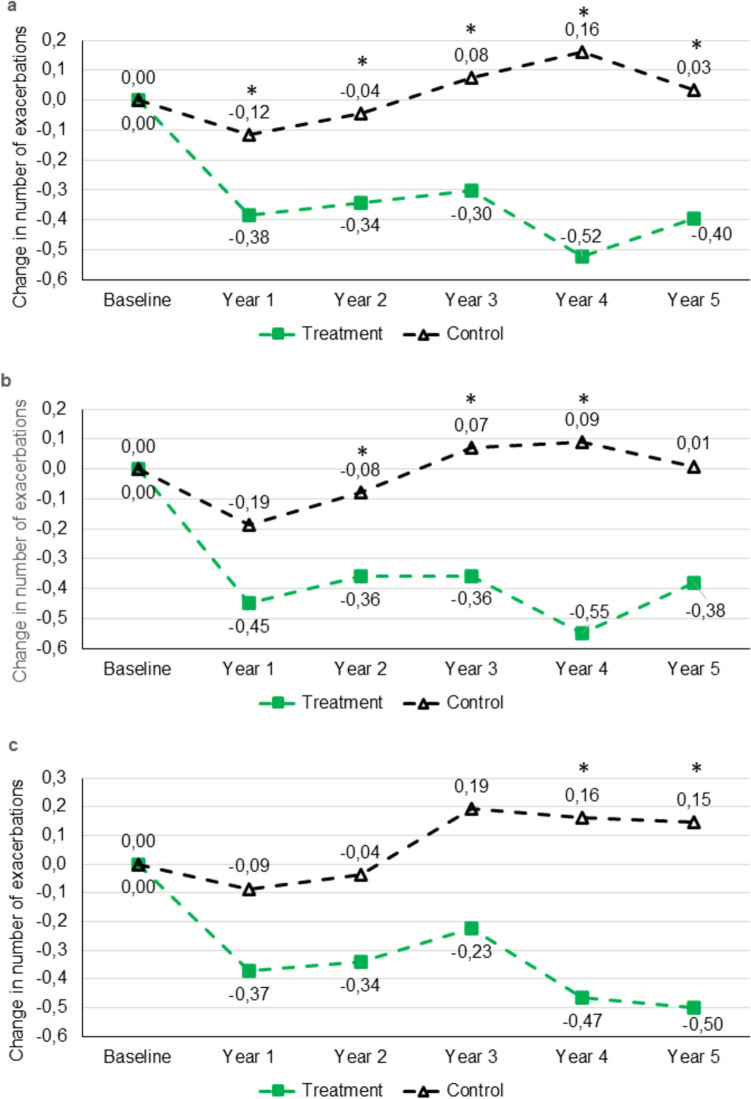
Moderate exacerbations in COPD patients treated with mucoactive drugs for 24 months (treatment) vs. no mucoactive treatment (control). Mean change from baseline in number of exacerbations for** a** all patients, **b** patients with cough at baseline, and **c** patients with severe/very severe COPD (GOLD stages 3‒4) at baseline

Moderate exacerbations of COPD in all patients were reduced to a significantly greater extent in the treatment vs. control cohort in all 5 years of follow-up (Table 5, Fig. 5a). This reduction of moderate exacerbations in the treatment cohort was also seen in the subgroups of patients with cough at baseline (Table 5, Fig. 5b) or COPD stages 3‒4 at baseline (Supplementary Table S11, Fig. 5c); the difference from the control cohort was significant in three or two years, respectively. The number of moderate exacerbations and change from baseline in the subgroups of patients with and without concurrent ICS use during the first two years of follow-up are shown in Supplementary Table S12.

**Table 5:** Moderate exacerbations in the treatment and control cohorts for all patients and those with cough at baseline

	All patients					Cough				
	*n*	Treatment cohort	*n*	Control cohort	*P* value	*n*	Treatment cohort	*n*	Control cohort	*P* value
Baseline	81	1.06 (1.09)	371	0.61 (1.31)	< 0.001*	69	1.15 (1.09)	252	0.74 (1.47)	< 0.001*
Year 1	81	0.68 (1.0)	368	0.50 (0.91)	0.128	69	0.70 (1.02)	250	0.56 (0.97)	0.342
Year 2	79	0.70 (1.02)	365	0.57 (1.17)	0.162	67	0.76 (1.07)	249	0.67 (1.33)	0.288
Year 3	66	0.68 (1.04)	307	0.66 (1.25)	0.450	56	0.75 (1.10)	210	0.78 (1.40)	0.629
Year 4	46	0.52 (0.78)	216	0.71 (1.04)	0.314	42	0.55 (0.80)	145	0.80 (1.11)	0.202
Year 5	38	0.58 (0.98)	174	0.60 (1.11)	0.729	34	0.65 (1.01)	112	0.69 (1.23)	0.719
Change from baseline										
Year 1	81	− 0.38 (1.17)	368	− 0.12 (1.24)	0.049*	69	− 0.45 (1.24)	250	− 0.19 (1.35)	0.082
Year 2	79	− 0.34 (0.99)	365	− 0.04 (1.26)	0.014*	67	− 0.36 (1.01)	249	− 0.08 (1.37)	0.043*
Year 3	66	− 0.30 (1.07)	307	0.08 (1.45)	0.008*	56	− 0.36 (1.12)	210	0.07 (1.60)	0.013*
Year 4	46	− 0.52 (1.19)	216	0.16 (1.19)	< 0.001*	42	− 0.55 (1.23)	145	0.09 (1.30)	0.002*
Year 5	38	− 0.40 (1.08)	174	0.03 (1.62)	0.015*	34	− 0.38 (1.10)	112	0.01 (1.78)	0.056

Data presented as mean (SD)

Only patients who had no missing data on exacerbations are included in the table

^*^Statistically significant difference between treatment and control cohorts

Exacerbation rates for patients with severe exacerbations of COPD, for those with GOLD stage 2 at baseline, and for the subgroups with a bronchitic, frequent exacerbator, or bronchiectasis-COPD overlap phenotypes at baseline are given in the Supplementary Tables S13‒S17. All patients, patients with cough at baseline and patients with GOLD stage 3 or 4 at baseline on mucoactive treatment had a greater reduction in severe exacerbations than controls, but the differences did not reach statistical significance, except for a significantly larger reduction in exacerbations with the patients with GOLD stage 3 or 4 in year 5. Patients with GOLD stage 2 at baseline with mucoactive treatment had a greater reduction in all and moderate exacerbations than controls, but due to the low number of patients in the treatment cohort, the differences did not reach statistical significance. Patients with a bronchitic phenotype had a greater reduction in all and moderate exacerbations in the treatment cohort than controls, with the differences being statistically significant at two years. In patients with an overlap of bronchiectasis and COPD, there was a numerical reduction in all, moderate and severe exacerbations in the treatment cohort vs. controls, but the differences were not statistically significant.

## Discussion

This real-world study with 5 years of follow-up, showed a significantly larger reduction from baseline in exacerbation rate in patients treated with mucoactive therapy for 24 months compared to the control group receiving standard of care only; after the completion of the two-year treatment period, most patients remained on their original treatment. This reduction in exacerbation rate was statistically significant throughout all 5 years of follow-up.

Our study population (*n* = 452) was a similar size to that of the RESTORE study (*n* = 467), although the cohort who received mucoactive treatment (*n* = 81) was smaller than the erdosteine group (*n* = 228) in the RESTORE study [8]. Most of the baseline characteristics differed between patients in the treatment and control cohorts of our real-world study, which contrasts with the carefully selected and matched patient samples in randomized controlled trials. A multivariate adjustment and sensitivity analysis were performed to control for baseline differences in the treatment and control cohorts and it showed consistency of results despite baseline differences. At baseline, patients in the treatment group had a significantly higher prevalence of cough and expectoration, worse CAT and FEV_1_, a higher frequency of exacerbations, and were more likely to have the bronchitic, frequent exacerbator, or bronchiectasis-COPD overlap phenotype than controls. These observations may be because patients in a worse condition before enrollment were more likely to be treated with mucoactive therapy after study enrollment in line with the concept of clinical phenotypes and treatable traits [17, 18, 20, 21]. Thus, in a real-world setting using the treatable traits-based approach, long-term mucoactive treatment was used more frequently in patients with bronchitic and exacerbation phenotypes and in clinically worse patients. A multivariate adjustment was performed to control for baseline disparities of treatment and control cohort.

Mucoactive therapy for 24 months on top of standard of care resulted in a significantly greater reduction from baseline in the rate of all exacerbations across all five years of follow-up compared to standard of care alone (controls). The RESTORE study, which followed patients for one year only, also demonstrated a significant reduction in the overall exacerbation rate in patients treated with erdosteine versus placebo [8]. Furthermore, the RESTORE study included patients with two or more exacerbations in the 12 months before study entry, whereas only 48.1% of patients in our study had two or more exacerbations in the 12 months before enrollment and 51.9% of patients had a history of none or only one exacerbation in the previous 12 months.

The treatable traits-based approach, as used in the Czech Republic, indicates the use of mucoactive treatment in patients with a bronchitic phenotype. In our study, 79% of patients in the treatment cohort had a bronchitic phenotype and 85.2% had a productive cough. As both our study and RESTORE demonstrated a reduction of exacerbations in the mucoactive-treated group, these findings suggest that mucoactive treatment leads to a reduction of exacerbations not only in patients with ≥ 2 exacerbations but also in patients with a bronchitic phenotype, regardless of the number of exacerbations in the previous year.

Our analysis of the subgroup of patients with severe and very severe COPD (GOLD stages 3‒4) found that mucoactive treatment reduced the rate of exacerbations compared to the control group, with the differences being statistically significant in years 2‒5. These results suggest a beneficial effect of mucoactive treatment on reducing exacerbations, even in patients with the most severe COPD, where exacerbations may have a substantial impact on health status. In this respect, our results differ from those of the post hoc analysis of the RESTORE study, which failed to show a significant difference in the exacerbation rate between the erdosteine-treated and control groups in the subgroup of patients with stage 3 COPD [9]. This may be due to the different clinical characteristics of the patients in the two studies: while 57% of patients in the RESTORE study were in stage 2 COPD, 43% were in stage 3 COPD, and none were in stage 4, the patients in our study had more severe disease, with 52.7% of patients in stage 3 or 4 COPD, and none of the stage 2 patients had a FEV_1_ > 60%. Nonetheless, our results support the conclusions from the RESTORE study that mucoactive treatment reduces the number of exacerbations in patients with COPD when added to standard of care. Additionally, our results suggest that this beneficial effect of mucoactive drugs also applies to patients with severe and very severe COPD.

The most recent GOLD report stated that regular treatment with mucoactive drugs may reduce exacerbations and modestly improve health status in COPD patients not receiving ICS [1]. This report mentioned that erdosteine may have a significant effect on (mild) exacerbations irrespective of concurrent treatment with ICS [1]. Oxidative stress drives chronic inflammation and is markedly increased in patients with COPD, especially during acute exacerbations, and contributes to the pathology of the disease. Corticosteroids are currently the main class of anti-inflammatory drugs used in the treatment of COPD to prevent exacerbations. However, oxidative stress may reduce corticosteroid sensitivity in COPD. Thiol-based mucolytic agents act as antioxidants and, in addition, may increase sensitivity to glucocorticoids. Few experimental studies have compared the effects of corticosteroids and thiol agents on oxidative stress. Some of these studies have found a better antioxidant effect of corticosteroids and other studies have shown a better effect of thiols. Other studies showed some evidence for greater antioxidant effects when thiols and corticosteroids are administered together [22].

Patients in our study with concurrent ICS treatment during the first 24 months were more symptomatic and had more exacerbations in the previous year than patients without concurrent ICS treatment. Our analyses of the subgroups of patients with and without concurrent ICS during the first 24 months of treatment found that the greater reduction in exacerbation rate in the treatment vs. controls was present in both subgroups with and without ICS use. These results support the hypothesis that the reduction in exacerbations was due to a direct effect of mucoactive treatment and not the result of treatment with an ICS. Furthermore, the effect of mucoactive treatment on the reduction of exacerbations was not affected by concomitant use of ICS consistent with the findings of the RESTORE study [6, 8, 22]. Our finding of an independent positive effect of mucoactive treatment on the incidence of exacerbations is not in contradiction with the latest GOLD 2025, erdosteine constitutes the majority of mucoactive medication in our cohort. The erdosteine data differ from those of N-acetylcysteine and carbocysteine, for which an effect was demonstrated in ICS naive subjects [1].

Patients who used mucoactive treatment and concomitant ICS during the first 24 months of treatment had a greater reduction in exacerbations than patients who received mucoactive treatment without ICS, although these differences did not reach statistical significance. These results may suggest a synergistic effect of mucoactive drugs and ICS on COPD exacerbations, but further research is needed.

Our findings of a significant reduction in moderate exacerbations in the cohort treated with mucoactive drugs vs. the controls support the results of the post hoc analysis of the RESTORE study [8, 10]. While the frequency of severe exacerbations was reduced more in the mucoactive treatment group and its subgroups compared with the control group and its subgroups, the differences did not reach statistical significance.

Chronic cough, a common symptom in COPD and potential predictor of acute exacerbations [23, 24], was present at baseline in 85.2% of the treatment cohort and 67.9% of the control cohort. Among patients with cough at baseline, there was a greater reduction in the exacerbation rate in the mucoactive-treated cohort compared to the controls, with significant differences between the groups at the end of years 2, 3, and 4. These results suggest that the presence of chronic cough may be a good predictor of the ability of mucoactive treatment to reduce the frequency of exacerbations.

Mucus hypersecretion was shown to be an important feature and independent risk factor for disease progression in a large observational study of patients with COPD [25]. Airway-occluding mucus plugs caused a rapid decline in lung function, deterioration of quality of life, higher risk of infections and pneumonia, a high rate of acute exacerbations, hospitalization, and mortality [25]. Thus, chronic bronchitis and chronic sputum production are treatable traits present in many patients with COPD [21]. Our observations of a larger reduction in exacerbation rate in the treatment cohort vs. controls in the subgroup with the bronchitic phenotype support earlier use of mucoactive drugs to target these important treatable traits. In patients with an overlap of bronchiectasis and COPD, there was a numerical reduction in exacerbations, but the differences were not statistically significant. This may be due to the small number of patients and the lack of power to detect this difference (effect) as statistically significant. In patients with frequent exacerbations, mucoactive treatment had no effect at all on the exacerbation rate. This may be influenced by the fact that we did not assess subtypes of exacerbations in our study. For example, in eosinophilic exacerbations, it is difficult to expect improvement after mucoactive medication.

Several issues are associated with the long-term duration of the real-life study. After 24 months, patients in both the treatment and control cohorts may or may not have been receiving mucoactive therapy. In years 3 to 5 of the study, 89.6% to 92.5% of patients in the treatment cohort remained on mucoactive therapy and 7.3% to 18.8% of patients in the control cohort subsequently received mucoactive therapy (Supplementary Table S6). Our results suggest that in real life, continuous mucoactive therapy leads to a reduction in exacerbations over 5 years, although a minority of patients changed therapy in years 3 to 5. Over the 5 years of the study, slightly more than half of the patients dropped out. This is probably related to the fact that patients with more advanced COPD with post-bronchodilator FEV_1_ ≤ 60% of predicted value were included, the average FEV_1_ value in the entire group was 46.7%. However, the proportion of patients that dropped out in the treatment and control groups was similar (Fig. 1). Patients who dropped out during the study were in worse condition at baseline than patients with completed follow-up, having worse CAT, lower FEV_1_ and BMI, had more exacerbations and had more often a frequent exacerbator phenotype and pulmonary cachexia phenotype (Supplementary Table S4). Among the patients who dropped out, some patients were lost to follow-up (17.3% and 26.1% in treatment and control cohort, respectively) and some patients died (33.3% and 25.1% in treatment and control cohort, respectively). The number of deaths during the study in the treatment cohort was slightly higher than in the control cohort and the difference did not reach statistical significance. Causes of death during the study did not differ significantly between the treatment and control groups (Supplementary Table S5).

## Strengths and Limitations

The strengths of our study are that it was a relatively large, prospective, real-life study involving 452 patients with COPD (FEV_1_ ≤ 60% of predicted) and no other restrictions on patient selection and inclusion, thereby reflecting routine clinical practice conditions. Also, this was a long-term study, monitoring COPD exacerbations over 5 years. However, a limitation of this real-life study was that it did not conform to the strict criteria required for a randomized controlled trial. In this real-life study most of the baseline characteristics differed between patients in the treatment and control cohort, and therefore a multivariate adjustment was performed to control for these baseline disparities. Furthermore, roughly half of the patients dropped out during the study. Finally, we did not perform Bonferroni correction or other adjustments to counteract the multiple comparisons problem.

## Conclusion

Overall, this real-world observational study showed that mucoactive treatment for two years in addition to usual care reduced the number of COPD exacerbations (all, moderate) in patients followed up for a further 3 years. The reduction in exacerbations was more pronounced in patients with cough and in patients with stage 3 to 4 COPD, but importantly was independent of the use of ICS.

Our results support the findings from randomized clinical trials and suggest that early use of mucoactive drugs in patients with COPD may be of value in the real world for reducing exacerbations, irrespective of concomitant use of ICS. Mucoactive drugs may be beneficial particularly in patients with cough and sputum production, with or without frequent exacerbations. In addition, our results support the importance of mucoactive treatment as part of a strategy to address treatable traits.

## Supplementary Information

Below is the link to the electronic supplementary material.

Supplementary file1 (PDF 304 KB)

## Data Availability

The data that supported the findings of this study are available from [Institute of Biostatistics and Analyses, Masaryk University, Brno, Czech Republic], but restrictions apply to the availability of these data, which were used under license for the current study and are not publicly available. Data are, however, available from the authors upon reasonable request and with the permission of [Institute of Biostatistics and Analyses, Masaryk University, Brno, Czech Republic]. Raw data for dataset are not publicly available to preserve individuals’ privacy under the European General Data Protection Regulation. If somebody wants to request the data from this study, please contact Katerina Kusalova (kusalova@biostatistika.cz) from Institute of Biostatistics and analyses. The institute will share the required dataset with her/his.
