# Supplementary material for: Effect of Treatment with Mucoactive Drugs on COPD Exacerbations During 5 years of Follow-up in the Czech Republic: A Real-World Study
Source: Lung. 2025 May 6;203(1):61. doi: 10.1007/s00408-025-00813-7 (PMC12055937; doi:10.1007/s00408-025-00813-7)
Supplement: Supplementary file 1 — Supplementary file1 (PDF 304 KB) [file 408_2025_813_MOESM1_ESM.pdf]

Supplementary Information

Effect of Treatment with Mucoactive Drugs on COPD Exacerbations During 5 years of Follow-up in the Czech Republic: A Real-World Study

Jaromír Zatloukal, Clive Page, Kristián Brat, Michal Svoboda, Eva Voláková, Marek Plutinský, Michal Kopecký, Vladimír Koblížek

Original research article for *Lung*

Corresponding author: Vladimir Koblizek, Faculty of Medicine in Hradec Kralove, Charles University, Czech Republic

email: vladimir.koblizek@fnhk.cz

Supplementary Table S1. Baseline demographic and clinical characteristics – comparison of 784 patients from the Czech Multicenter Research Database of COPD (CMRDC) included into the study and patients excluded from the study (see Figure 1 in main text)

		Included patients (n=452)	Excluded patients (n=352)	P-value
Male	N (%)	331 (73.2)	241 (72.6)	0.842
Age (years)	Mean (SD)	65.7 (9.5)	67.7 (8.8)	0.013*
Smoking status	Current smoker, n (%)	87 (19.2)	73 (22.0)	0.593
	Ex-smoker, n (%)	313 (69.2)	225 (67.8)	
	Non-smoker, n (%)	52 (11.5)	34 (10.2)	
BMI (kg/m^2)	Mean (SD)	28.4 (6.2)	26.1 (5.9)	<0.001*
Dyspnea (mMRC)	Mean (SD)	2.2 (1.0)	2.3 (1.1)	0.207
CAT score	Mean (SD)	14.9 (7.5)	17.6 (7.8)	<0.001*
Chronic cough	N (%)	321 (71.0)	242 (72.9)	0.564
Expectoration	N (%)	253 (56.0)	202 (60.8)	0.172
FEV_1 (% predicted)	Mean (SD)	46.7 (11.6)	42.6 (11.4)	<0.001*
GOLD stage[a], n (%)	2	172 (42.0)	95 (30.4)	0.002*
	3	196 (47.8)	166 (53.2)	
	4	42 (10.2)	51 (16.3)	
	Unknown	42	20	
Clinical phenotype	Bronchitic	253 (56.0)	202 (60.8)	0.172
	Emphysematic	165 (73.7)	125 (81.2)	0.090
	BCO	61 (27.4)	52 (35.4)	0.101
	ACO	17 (4.7)	6 (2.4)	0.132

	Frequent exacerbator	113 (25.0)	132 (39.8)	<0.001*
	Pulmonary cachexia	42 (9.3)	69 (20.8)	<0.001*
Exacerbations in previous 12 months, mean (SD)	All	1.0 (1.5)	1.4 (1.7)	<0.001*
	Moderate	0.7 (1.3)	0.9 (1.4)	0.010*
	Severe	0.3 (0.7)	0.5 (0.9)	<0.001*
Frequency of all exacerbations in previous 12 months, n (%)	0	234 (51.8)	138 (41.6)	<0.001*
	1	105 (23.2)	62 (18.7)	
	2	54 (11.9)	62 (18.7)	
	3	29 (6.4)	32 (9.6)	
	>3	30 (6.6)	38 (11.4)	
Treatment at baseline, n (%)	Containing ICS	241 (53.3)	172 (51.8)	0.675
	Containing LABA	386 (85.4)	295 (88.9)	0.157
	Containing LAMA	324 (71.7)	260 (78.3)	0.035*
	LAMA + LABA	171 (37.8)	149 (44.9)	0.047*
	LAMA+LABA+ICS	183 (40.5)	137 (41.3)	0.827
	Erdosteine	61 (13.5)	77 (23.2)	<0.001*
	N-acetylcysteine	3 (0.7)	10 (3.0)	0.011*
	Theophylline	208 (46.0)	169 (50.9)	0.176
	Roflumilast	45 (10.0)	42 (12.7)	0.235

ACO Asthma-COPD overlap, BCO bronchiectasis with COPD, BMI Body Mass Index, CAT COPD Assessment Test (score range 0-40), FEV_1 Forced Expiratory Volume in one second, ICS Inhaled corticosteroid, LABA long-acting muscarinic antagonist, LAMA long-acting $beta_2$-agonist, mMRC modified Medical Research Council dyspnea scale

[a]GOLD stage 2, 50% ≤ FEV_1 < 80% predicted; GOLD stage 3, 30% ≤ FEV_1 < 50% predicted; GOLD stage 4, FEV_1 < 30% predicted.

*Statistically significant difference between included and excluded patients

Supplementary Table S2. Univariate and multivariate linear model with mixed effects to control for baseline disparities

Table S2a: Linear model with mixed effects – univariate

*Number of all exacerbations during 5 years ~ cohort * year, random = year | ID*

	Coeff	95% CI	p-value
Intercept[1]	0.737	(0.612; 0.861)	<0.001
Treatment cohort[2]	0.685	(0.390; 0.980)	<0.001
Year[3]	0.048	(0.010; 0.085)	0.013
Treatment cohort * Year[4]	-0.172	(-0.262; -0.082)	<0.001

[1] Mean of number of exacerbations in Control cohort at time 0 (baseline) = 0.74.

[2] Difference between treatment and control cohort at time 0 (baseline) = 0.69. It means that mean of number of exacerbations in Treatment cohort at time 0 (baseline) is 0.74+0.69 = 1.43.

[3] Change of number of exacerbations per 1 year in Control cohort = 0.05.

[4] Difference between treatment cohort and control cohort in change of number of exacerbations per 1 year = -0.17. It means that change of number of exacerbations per 1 year in Treatment cohort is 0.05-0.17 = -0.12.

Table S2b: Linear model with mixed effects – multivariate (adjusted by baseline FEV_1 (%), cough, and number of exacerbations at baseline)

*Number of all exacerbations during 5 years ~ cohort * year + FEV_1 + cough + baseline number of exacerbations, random = year | ID*

	Coeff	95% CI	p-value
Intercept[1]	0.303	(0.031; 0.576)	0.029
Treatment cohort[2]	0.177	(0.006; 0.349)	0.042
Year[3]	0.061	(0.018; 0.105)	0.006
Treatment cohort * Year[4]	-0.186	(-0.289; -0.082)	<0.001
FEV_1	-0.003	(-0.008; 0.002)	0.255
Cough	0.018	(-0.112; 0.149)	0.779
Number of baseline exacerbations	0.643	(0.604; 0.682)	<0.001

[1] Mean of number of exacerbations in Control cohort at time 0 (baseline) = 0.30.

[2] Difference between treatment and control cohort at time 0 (baseline) = 0.18. It means that mean of number of exacerbations in Treatment cohort at time 0 (baseline) is 0.30+0.18 = 0.48.

[3] Change of number of exacerbations per 1 year in Control cohort = 0.06.

[4] Difference between treatment cohort and control cohort in change of number of exacerbations per 1 year = -0.19. It means that change of number of exacerbations per 1 year in Treatment cohort is 0.06-0.19 = -0.13.

Table S2c: Linear model with mixed effects – multivariate (adjusted by baseline FEV_1 (%), cough, number of exacerbations, and ICS use at baseline)

*Number of all exacerbations during 5 years ~ cohort * year + FEV_1 + cough + baseline number of exacerbations + ICS use during first 24 months, random = year | ID*

	Coeff	95% CI	p-value
Intercept[1]	0.291	(-0.007; 0.590)	0.055
Treatment cohort[2]	0.177	(0.000; 0.354)	0.051
Year[3]	0.056	(0.012; 0.100)	0.013
Treatment cohort * Year[4]	-0.194	(-0.301; -0.088)	<0.001
FEV_1	-0.004	(-0.009; 0.002)	0.187
Cough	0.038	(-0.098; 0.173)	0.586
Number of baseline exacerbations	0.638	(0.597; 0.679)	<0.001
ICS use during first 24 months	0.083	(-0.042; 0.209)	0.193

[1] Mean of number of exacerbations in Control cohort at time 0 (baseline) = 0.29.
[2] Difference between treatment and control cohort at time 0 (baseline) = 0.18. It means that mean of number of exacerbations in Treatment cohort at time 0 (baseline) is 0.29+0.18 = 0.47.
[3] Change of number of exacerbations per 1 year in Control cohort = 0.06.
[4] Difference between treatment cohort and control cohort in change of number of exacerbations per 1 year = -0.19. It means that change of number of exacerbations per 1 year in Treatment cohort is 0.06-0.19 = -0.13.

Supplementary Table S3. Sensitivity analysis - Linear models with mixed effects

Table S3a: Linear model with mixed effects – prediction of number of **all** exacerbations

*Number of all exacerbations during 5 years ~ cohort * year, random = year | ID*

	Coeff	95% CI	p-value
Treatment cohort * Year[1]	-0.172	(-0.262; -0.082)	<0.001
Adjusted by FEV_1	-0.173	(-0.263; -0.083)	<0.001
Adjusted by cough	-0.173	(-0.263; -0.083)	<0.001
Adjusted by number of baseline exacerbations (all)	-0.185	(-0.288; -0.083)	<0.001
Adjusted by FEV_1 and cough	-0.174	(-0.264; -0.084)	<0.001
Adjusted by FEV_1 and number of baseline exacerbations (all)	-0.185	(-0.287; -0.083)	<0.001
Adjusted by cough and number of baseline exacerbations (all)	-0.185	(-0.288; -0.083)	<0.001
Adjusted by FEV_1 and cough and number of baseline exacerbations (all)	-0.186	(-0.289; -0.082)	<0.001

[1] **Univariate analysis**. Difference between the treatment cohort and the control cohort in change of number of exacerbations per 1 year = -0.17. On average, the number of exacerbations decreased by 0.17 more in the treatment cohort than in the control cohort.

Similar results are seen after adjusting by FEV_1, cough and baseline number of exacerbations. Results are consistent in this sensitivity analysis.

Table S3b: Linear model with mixed effects – prediction of number of **moderate** exacerbations

*Number of moderate exacerbations during 5 years ~ cohort * year, random = year | ID*

	Coeff	95% CI	p-value
Treatment cohort * Year[1]	-0.123	(-0.193; -0.053)	<0.001
Adjusted by FEV_1	-0.123	(-0.193; -0.053)	<0.001
Adjusted by cough	-0.124	(-0.194; -0.053)	<0.001
Adjusted by number of baseline exacerbations (moderate)	-0.132	(-0.212; -0.051)	0.001
Adjusted by FEV_1 and cough	-0.124	(-0.195; -0.054)	<0.001
Adjusted by FEV_1 and number of baseline exacerbations (moderate)	-0.131	(-0.212; -0.051)	0.001
Adjusted by cough and number of baseline exacerbations (moderate)	-0.131	(-0.212; -0.051)	0.001
Adjusted by FEV_1 and cough and number of baseline exacerbations (moderate)	-0.131	(-0.211; -0.051)	0.001

[1] **Univariate analysis**. Difference between the treatment cohort and the control cohort in change of number of exacerbations per 1 year = -0.12. On average, the number of exacerbations decreased by 0.12 more in the treatment cohort than in the control cohort.

Similar results are seen after adjusting by FEV_1, cough and baseline number of exacerbations. Results are consistent in this sensitivity analysis.

Table S3c: Linear model with mixed effects – prediction of number of **severe** exacerbations

*Number of severe exacerbations during 5 years ~ cohort * year, random = year | ID*

	Coeff	95% CI	p-value
Treatment cohort * Year[1]	-0.048	(-0.097; 0.002)	0.059
Adjusted by FEV_1	-0.049	(-0.098; 0.001)	0.055
Adjusted by cough	-0.048	(-0.098; 0.002)	0.058
Adjusted by number of baseline exacerbations (severe)	-0.053	(-0.115; 0.008)	0.091
Adjusted by FEV_1 and cough	-0.049	(-0.098; 0.001)	0.054
Adjusted by FEV_1 and number of baseline exacerbations (severe)	-0.053	(-0.115; 0.009)	0.091
Adjusted by cough and number of baseline exacerbations (severe)	-0.053	(-0.115; 0.009)	0.091
Adjusted by FEV_1 and cough and number of baseline exacerbations (severe)	-0.053	(-0.115; 0.009)	0.091

[1] **Univariate analysis**. Difference between the treatment cohort and the control cohort in change of number of exacerbations per 1 year = -0.05. On average, the number of exacerbations decreased by 0.05 more in the treatment cohort than in the control cohort.

Similar results are seen after adjusting by FEV_1, cough and baseline number of exacerbations. Results are consistent in this sensitivity analysis.

Supplementary Table S4. Baseline demographic and clinical characteristics of patients who dropped out during the study and remaining patients with completed 5-year follow-up

		Dropped patients (N=231)	Remaining patients (N=221)	P-value
Male	N (%)	167 (72.3)	164 (74.2)	0.842
Age (years)	Mean (SD)	67.1 (9.0)	64.4 (9.8)	0.013*
Smoking status	Current smoker, n (%)	52 (22.5)	35 (15.8)	0.593
	Ex-smoker, n (%)	154 (66.7)	159 (71.9)	
	Non-smoker, n (%)	25 (10.8)	27 (12.2)	
BMI (kg/m^2)	Mean (SD)	28.3 (6.8)	28.6 (5.4)	<0.001*
Dyspnea (mMRC)	Mean (SD)	2.4 (1.0)	2.0 (1.0)	0.207
CAT score	Mean (SD)	15.1 (7.4)	14.7 (7.6)	<0.001*
Chronic cough	N (%)	171 (74.0)	150 (67.9)	0.564
Expectoration	N (%)	122 (52.8)	131 (59.3)	0.172
FEV_1 (% predicted)	Mean (SD)	46.8 (11.7)	46.5 (11.4)	<0.001*
Clinical phenotype	Bronchitic	122 (52.8)	131 (59.3)	0.172
	Emphysematic	70 (76.9)	95 (71.4)	0.090
	BCO	23 (25.3)	38 (28.8)	0.101
	ACO	9 (4.9)	8 (4.6)	0.132
	Frequent exacerbator	64 (27.7)	49 (22.2)	<0.001*
	Pulmonary cachexia	29 (12.6)	13 (5.9)	<0.001*
Exacerbations in previous 12 months, mean (SD)	All	1.1 (1.6)	0.9 (1.5)	<0.001*
	Moderate	0.7 (1.3)	0.6 (1.3)	0.010*
	Severe	0.3 (0.7)	0.3 (0.7)	<0.001*
Frequency of all exacerbations in previous 12 months, n (%)	0	109 (47.2)	125 (56.6)	<0.001*
	1	58 (25.1)	47 (21.3)	
	2	28 (12.1)	26 (11.8)	
	3	20 (8.7)	9 (4.1)	
	>3	16 (6.9)	14 (6.3)	

Treatment at baseline, n (%)	Containing ICS	118 (51.1)	123 (55.7)	0.675
	Containing LABA	185 (80.1)	201 (91.0)	0.157
	Containing LAMA	157 (68.0)	167 (75.6)	0.035*
	LAMA + LABA	82 (35.5)	89 (40.3)	0.047*
	LAMA+LABA+ICS	84 (36.4)	99 (44.8)	0.827
	Erdosteine	29 (12.6)	32 (14.5)	<0.001*
	N-acetylcysteine	1 (0.4)	2 (0.9)	0.011*
	Theophylline	109 (47.2)	99 (44.8)	0.610
	Roflumilast	22 (9.5)	23 (10.4)	0.754

ACO Asthma-COPD overlap, BCO bronchiectasis with COPD, BMI Body Mass Index, CAT COPD Assessment Test (score range 0-40), FEV_1 Forced Expiratory Volume in one second, ICS Inhaled corticosteroid, LABA long-acting muscarinic antagonist, LAMA long-acting $beta_2$-agonist, mMRC modified Medical Research Council dyspnea scale

*Statistically significant difference between ICS use and non-ICS use cohorts

Supplementary Table S5. Causes of death during the study – comparison of treatment and control cohorts.

		All (n=452)	Treatment cohort (n=81)	Control cohort (n=371)	P-value
Death	N (%)	120 (26.5)	27 (33.3)	93 (25.1)	0.127
Cause of death, n (%)	Respiratory	52 (43.3)	11 (40.7)	41 (44.1)	0.542
	Cardiovascular	29 (24.2)	9 (33.3)	20 (21.5)	
	Cancer	28 (23.3)	4 (14.8)	24 (25.8)	
	Other	8 (6.7)	2 (7.4)	6 (6.5)	
	Covid	3 (2.5)	1 (3.7)	2 (2.2)	

Supplementary Table S6. Number of patients with mucoactive treatment during the treatment and follow-up periods

	Treatment cohort n = 81	Control cohort n = 371
1 year		
Without mucoactive drugs	0 (0.0)	371 (100.0)
With mucoactive drugs	81 (100.0)	0 (0.0)
2 years		
Without mucoactive drugs	0 (0.0)	371 (100.0)
With mucoactive drugs	81 (100.0)	0 (0.0)
3 years		
Without mucoactive drugs	6 (9.2)	290 (92.7)
With mucoactive drugs	59 (90.8)	23 (7.3)
4 years		
Without mucoactive drugs	5 (10.4)	200 (89.7)
With mucoactive drugs	43 (89.6)	23 (10.3)
5 years		
Without mucoactive drugs	3 (7.5)	147 (81.2)
With mucoactive drugs	37 (92.5)	34 (18.8)

Data presented as number (percentage)

Supplementary Table S7. Number of patients with inhaled corticosteroids (ICS) during the treatment and follow-up periods

	Treatment cohort n = 81	Control cohort n = 371
1 year		
Without ICS	31 (38.3)	184 (49.6)
With ICS	50 (61.7)	187 (50.4)
2 years		
Without ICS	31 (38.3)	178 (48.0)
With ICS	50 (61.7)	193 (52.0)
3 years		
Without ICS	27 (41.5)	155 (49.5)
With ICS	38 (58.5)	158 (50.5)
4 years		
Without ICS	23 (47.9)	110 (49.3)
With ICS	25 (52.1)	113 (50.7)
5 years		
Without ICS	20 (50.0)	86 (47.5)
With ICS	20 (50.0)	95 (52.5)

Data presented as number (percentage)

Supplementary Table S8. Baseline characteristics of patient subgroups by phenotype

		Bronchitic phenotype			Frequent exacerbator phenotype			BCO phenotype		
		Treatment cohort	Control cohort	P value	Treatment cohort	Control cohort	P value	Treatment cohort	Control cohort	P value
Male	N (%)	47 (73.4)	145 (76.7)	0.596	28 (71.8)	53 (71.6)	0.984	12 (66.7)	32 (74.4)	0.538
Age (years)	Mean (SD)	66.7 (7.6)	65.6 (9.9)	0.609	66.9 (6.7)	66.2 (9.9)	0.940	65.0 (5.0)	66.2 (9.8)	0.447
Smoking status	Current smoker, n (%)	14 (21.9)	48 (25.4)	0.748	6 (15.4)	11 (14.9)	>0.999	4 (22.2)	8 (18.6)	0.921
	Ex-smoker, n (%)	44 (68.8)	120 (63.5)		30 (76.9)	56 (75.7)		12 (66.7)	28 (65.1)	
	Non-smoker, n (%)	6 (9.4)	21 (11.1)		3 (7.7)	7 (9.5)		2 (11.1)	7 (16.3)	
BMI (kg/m^2)	Mean (SD)	28.0 (5.2)	28.7 (6.5)	0.726	28.9 (4.4)	27.2 (5.0)	0.065	27.9 (4.0)	26.2 (5.2)	0.186
Dyspnea (mMRC)	Mean (SD)	2.3 (1.1)	2.2 (0.9)	0.329	2.6 (1.1)	2.6 (1.1)	0.960	1.9 (1.1)	1.9 (1.1)	0.947
CAT score	Mean (SD)	19.2 (7.0)	15.8 (7.5)	<0.001*	20.6 (6.6)	19.3 (7.4)	0.339	17.2 (7.7)	16.0 (8.2)	0.576
Chronic cough	N (%)	63 (98.4)	174 (92.1)	0.080	36 (92.3)	57 (77.0)	0.043*	16 (88.9)	28 (65.1)	0.059
Expectoration	N (%)	64 (100.0)	189 (100.0)	-	32 (82.1)	42 (56.8)	0.007*	16 (88.9)	22 (51.2)	0.006*
FEV_1 (% predicted)	Mean (SD)	42.4 (11.7)	46.8 (10.8)	0.019*	41.3 (12.3)	47.1 (12.9)	0.021*	42.4(11.3)	45.3 (12.0)	0.419
Clinical phenotype	Bronchitic	64 (100.0)	189 (100.0)	-	32 (82.1)	42 (56.8)	0.007*	16 (88.9)	22 (51.2)	0.006*
	Emphysematic	28 (71.8)	68 (73.1)	0.876	18 (81.8)	27 (79.4)	>0.999	14 (77.8)	31 (72.1)	0.757
	BCO	16 (41.0)	22 (23.9)	0.048*	9 (40.9)	10 (29.4)	0.375	18 (100.0)	43 (100.0)	-
	ACO	0 (0.0)	10 (6.9)	0.122	0 (0.0)	4 (6.8)	0.294	0 (0.0)	1 (2.9)	>0.999
	Frequent exacerbator	32 (50.0)	42 (22.2)	<0.001*	39 (100.0)	74 (100.0)	-	9 (50.0)	10 (23.3)	0.040*
	Pulmonary cachexia	7 (10.9)	14 (7.4)	0.376	1 (2.6)	7 (9.5)	0.259	1 (5.6)	8 (18.6)	0.259
Exacerbations in previous 12 months, mean (SD)	All	1.7 (1.5)	0.9 (1.5)	<0.001*	2.9 (0.9)	3.1 (2.1)	0.414	1.8 (1.7)	0.8 (1.4)	0.007*
	Moderate	1.1 (1.1)	0.7 (1.4)	<0.001*	1.9 (1.0)	2.4 (2.1)	0.485	1.1 (0.9)	0.6 (1.2)	0.016*
	Severe	0.6 (1.0)	0.2 (0.7)	<0.001*	1.1 (1.2)	0.8 (1.0)	0.163	0.8 (1.3)	0.2 (0.5)	0.051
Frequency of all	0	19 (29.7)	102 (54.0)		0 (0.0)	0 (0.0)		5 (27.8)	29 (67.4)	
	1	13 (20.3)	45 (23.8)		0 (0.0)	0 (0.0)		4 (22.2)	4 (9.3)	

exacerbations in previous 12 months, n (%)	2	12 (18.8)	22 (11.6)	<0.001*	15 (38.5)	39 (52.7)	0.171	4 (22.2)	4 (9.3)	0.010*
	3	10 (15.6)	7 (3.7)		14 (35.9)	15 (20.3)		0 (0.0)	3 (7.0)	
	>3	10 (15.6)	13 (6.9)		10 (25.6)	20 (27.0)		5 (27.8)	3 (7.0)	
Treatment at baseline, n (%)	Containing ICS	41 (64.1)	98 (51.9)	0.090	29 (74.4)	52 (70.3)	0.647	11 (61.1)	24 (55.8)	0.703
	Containing LABA	62 (96.9)	160 (84.7)	0.010*	36 (92.3)	67 (90.5)	>0.999	18 (100.0)	40 (93.0)	0.548
	Containing LAMA	57 (89.1)	123 (65.1)	<0.001*	33 (84.6)	50 (67.6)	0.051	17 (94.4)	40 (93.0)	>0.999
	LAMA + LABA	36 (56.2)	63 (33.3)	0.001*	20 (51.3)	23 (31.1)	0.035*	13 (72.2)	23 (53.5)	0.175
	LAMA+LABA+ICS	38 (59.4)	68 (36.0)	0.001*	26 (66.7)	34 (45.9)	0.036*	11 (61.1)	22 (51.2)	0.477
	Erdosteine	47 (73.4)	3 (1.6)	<0.001*	31 (79.5)	1 (1.4)	<0.001*	13 (72.2)	2 (4.7)	<0.001*
	N-acetylcysteine	3 (4.7)	0 (0.0)	0.016*	1 (2.6)	0 (0.0)	0.345	0 (0.0)	0 (0.0)	-
	Theophylline	32 (50.0)	90 (47.6)	0.742	18 (46.2)	40 (54.1)	0.424	7 (38.9)	17 (39.5)	0.962
	Roflumilast	17 (26.6)	17 (9.0)	<0.001*	12 (30.8)	12 (16.2)	0.072	8 (44.4)	3 (7.0)	0.001*

ACO Asthma-COPD overlap, BCO bronchiectasis with COPD, BMI Body Mass Index, CAT COPD Assessment Test (score range 0-40), FEV_1 Forced Expiratory Volume in one second, ICS Inhaled corticosteroid, LABA long-acting muscarinic antagonist, LAMA long-acting $beta_2$-agonist, mMRC modified Medical Research Council dyspnea scale

*Statistically significant difference between treatment and control cohorts

Supplementary Table S9. Baseline demographic and clinical characteristics according to ICS use or no use for 24 months

		All patients			Treatment cohort			Control cohort		
		No ICS use during first 24 months (N=200)	ICS use during first 24 months (N=228)	P value	No ICS use during first 24 months (N=29)	ICS use during first 24 months (N=48)	P value	No ICS use during first 24 months (N=171)	ICS use during first 24 months (N=180)	P value
Male	N (%)	146 (73.0)	167 (73.2)	0.954	20 (69.0)	34 (70.8)	0.862	126 (73.7)	133 (73.9)	0.965
Age (years)	Mean (SD)	65.3 (9.3)	66.3 (9.6)	0.098	67.5 (6.8)	67.6 (7.2)	0.825	64.9 (9.6)	65.9 (10.2)	0.082
Smoking status	Current smoker, n (%)	54 (27.0)	24 (10.5)	<0.001*	9 (31.0)	5 (10.4)	0.049*	45 (26.3)	19 (10.6)	<0.001*
	Ex-smoker, n (%)	123 (61.5)	177 (77.6)		16 (55.2)	38 (79.2)		107 (62.6)	139 (77.2)	
	Non-smoker, n (%)	23 (11.5)	27 (11.8)		4 (13.8)	5 (10.4)		19 (11.1)	22 (12.2)	
BMI (kg/m^2)	Mean (SD)	29.0 (6.4)	28.2 (5.8)	0.340	29.1 (5.4)	27.7 (5.1)	0.326	29.0 (6.6)	28.3 (5.9)	0.542
Dyspnea (mMRC)	Mean (SD)	2.0 (1.0)	2.4 (1.1)	0.002*	1.8 (1.0)	2.6 (1.0)	0.001*	2.1 (1.0)	2.3 (1.1)	0.091
CAT score	Mean (SD)	13.5 (7.1)	16.1 (7.6)	<0.001*	18.3 (6.1)	19.3 (7.6)	0.493	12.7 (7.0)	15.3 (7.4)	<0.001*
Chronic cough	N (%)	147 (73.5)	155 (68.0)	0.211	26 (89.7)	39 (81.3)	0.518	121 (70.8)	116 (64.4)	0.207
Expectoration	N (%)	111 (55.5)	130 (57.0)	0.752	22 (75.9)	38 (79.2)	0.735	89 (52.0)	92 (51.1)	0.861
FEV_1 (% predicted)	Mean (SD)	48.0 (10.6)	45.7 (12.1)	0.043*	43.8 (9.6)	43.3 (12.7)	0.888	48.7 (10.6)	46.3 (12.0)	0.051
Clinical phenotype	Bronchitic	111 (55.5)	130 (57.0)	0.752	22 (75.9)	38 (79.2)	0.735	89 (52.0)	92 (51.1)	0.861
	Emphysematic	57 (72.2)	96 (72.7)	0.928	10 (76.9)	21 (72.4)	>0.999	47 (71.2)	75 (72.8)	0.820
	BCO	25 (31.6)	33 (25.2)	0.311	6 (46.2)	11 (37.9)	0.616	19 (28.8)	22 (21.6)	0.287
	ACO	0 (0.0)	16 (9.2)	<0.001*	0 (0.0)	0 (0.0)	-	0 (0.0)	16 (11.6)	<0.001*
	Frequent exacerbator	29 (14.5)	79 (34.6)	<0.001*	10 (34.5)	28 (58.3)	0.043*	19 (11.1)	51 (28.3)	<0.001*
	Pulmonary cachexia	16 (8.0)	20 (8.8)	0.774	2 (6.9)	4 (8.3)	>0.999	14 (8.2)	16 (8.9)	0.814
Exacerbations in previous 12	All	0.6 (1.0)	1.4 (1.9)	<0.001*	1.2 (1.4)	1.9 (1.4)	0.034*	0.5 (0.9)	1.2 (1.9)	<0.001*
	Moderate	0.4 (0.8)	1.0 (1.6)	<0.001*	1.0 (1.2)	1.2 (1.0)	0.404	0.3 (0.7)	0.9 (1.7)	<0.001*

months, mean (SD)	Severe	0.2 (0.5)	0.4 (0.8)	0.014*	0.2 (0.5)	0.8 (1.2)	0.028*	0.2 (0.5)	0.3 (0.7)	0.202
Frequency of all exacerbations in previous 12 months, n (%)	0	125 (62.5)	95 (41.7)		12 (41.4)	10 (20.8)		113 (66.1)	85 (47.2)	
	1	46 (23.0)	54 (23.7)		7 (24.1)	10 (20.8)		39 (22.8)	44 (24.4)	
	2	17 (8.5)	34 (14.9)	<0.001*	3 (10.3)	11 (22.9)	0.105	14 (8.2)	23 (12.8)	<0.001*
	3	7 (3.5)	21 (9.2)		6 (20.7)	8 (16.7)		1 (0.6)	13 (7.2)	
	>3	5 (2.5)	24 (10.5)		1 (3.4)	9 (18.8)		4 (2.3)	15 (8.3)	
Treatment at baseline, n (%)	Containing ICS	13 (6.5)	217 (95.2)	<0.001*	3 (10.3)	46 (95.8)	<0.001*	10 (5.8)	171 (95.0)	<0.001*
	Containing LABA	143 (71.5)	222 (97.4)	<0.001*	25 (86.2)	47 (97.9)	0.064	118 (69.0)	175 (97.2)	<0.001*
	Containing LAMA	128 (64.0)	181 (79.4)	<0.001*	24 (82.8)	44 (91.7)	0.285	104 (60.8)	137 (76.1)	0.002*
	LAMA + LABA	94 (47.0)	67 (29.4)	<0.001*	20 (69.0)	23 (47.9)	0.072	74 (43.3)	44 (24.4)	<0.001*
	LAMA+LABA+ICS	7 (3.5)	169 (74.1)	<0.001*	2 (6.9)	42 (87.5)	<0.001*	5 (2.9)	127 (70.6)	<0.001*
	Erdosteine	22 (11.0)	38 (16.7)	0.092	22 (75.9)	34 (70.8)	0.631	0 (0.0)	4 (2.2)	0.123
	N-acetylcysteine	1 (0.5)	1 (0.4)	>0.999	1 (3.4)	1 (2.1)	>0.999	0 (0.0)	0 (0.0)	-
	Theophylline	73 (36.5)	120 (52.6)	<0.001*	12 (41.4)	21 (43.8)	0.839	61 (35.7)	99 (55.0)	<0.001*
	Roflumilast	16 (8.0)	29 (12.7)	0.112	8 (27.6)	13 (27.1)	0.962	8 (4.7)	16 (8.9)	0.118

ACO Asthma-COPD overlap, BCO bronchiectasis with COPD, BMI Body Mass Index, CAT COPD Assessment Test (score range 0-40), FEV_1 Forced Expiratory Volume in one second, ICS Inhaled corticosteroid, LABA long-acting muscarinic antagonist, LAMA long-acting beta$_2$-agonist, mMRC modified Medical Research Council dyspnea scale

*Statistically significant difference between ICS use and non-ICS use cohorts

Supplementary Table S10. All exacerbations in the treatment cohort with vs. without ICS use in first 24 months and in the control cohort with vs. without ICS use in first 24 months

	Treatment cohort					Control cohort				
	n	ICS	n	No ICS	P value	n	ICS	n	No ICS	P value
Baseline	48	1.94 (1.45)	29	1.24 (1.38)	0.034*	180	1.20 (1.94)	171	0.50 (0.86)	<0.001*
Year 1	48	1.31 (1.39)	29	0.69 (1.17)	0.041*	179	0.84 (1.23)	170	0.55 (0.96)	0.029*
Year 2	47	1.32 (1.48)	28	0.64 (0.99)	0.03*	178	0.93 (1.48)	169	0.58 (1.26)	<0.001*
Year 3	38	1.24 (1.38)	24	0.67 (0.96)	0.113	150	0.97 (1.39)	142	0.71 (1.48)	0.009*
Year 4	24	1.00 (1.25)	18	0.78 (1.11)	0.515	103	1.04 (1.36)	105	0.69 (1.11)	0.027*
Year 5	18	1.00 (1.03)	16	0.75 (1.29)	0.296	91	0.91 (1.27)	77	0.84 (1.75)	0.162
Change from baseline										
Year 1	48	-0.63 (1.75)	29	-0.55 (1.48)	0.961	179	-0.37 (1.78)	170	0.05 (1.06)	0.042*
Year 2	47	-0.62 (1.65)	28	-0.54 (1.20)	0.853	178	-0.26 (1.68)	169	0.07 (1.35)	0.283
Year 3	38	-0.71 (1.63)	24	-0.38 (0.97)	0.559	150	-0.15 (1.71)	142	0.22 (1.48)	0.226
Year 4	24	-0.96 (2.01)	18	-0.39 (1.04)	0.423	103	-0.08 (1.70)	105	0.26 (1.25)	0.283
Year 5	18	-0.83 (1.65)	16	-0.38 (0.89)	0.325	91	-0.22 (2.07)	77	0.44 (1.94)	0.256

Data presented as mean (SD)
Only patients who had no missing data on exacerbations are included in the table
*Statistically significant difference between ICS use vs. no ICS use

Supplementary Table S11. Moderate exacerbations in the treatment and control cohorts for patients with GOLD stages 3 or 4 COPD at baseline

	GOLD Stage 3 or 4				
	n	Treatment cohort	n	Control cohort	P value
Baseline	51	1.08 (1.09)	187	0.68 (1.40)	0.001*
Year 1	51	0.71 (1.03)	186	0.60 (1.01)	0.559
Year 2	50	0.72 (1.09)	184	0.65 (1.34)	0.517
Year 3	40	0.85 (1.19)	155	0.83 (1.51)	0.439
Year 4	30	0.63 (0.81)	106	0.74 (1.01)	0.814
Year 5	22	0.68 (1.17)	89	0.72 (1.18)	0.920
Change from baseline					
Year 1	51	-0.37 (1.10)	186	-0.09 (1.19)	0.066
Year 2	50	-0.34 (1.04)	184	-0.04 (1.20)	0.119
Year 3	40	-0.23 (1.07)	155	0.19 (1.50)	0.077
Year 4	30	-0.47 (1.07)	106	0.16 (1.09)	0.003*
Year 5	22	-0.50 (1.14)	89	0.15 (1.48)	0.038*

Data presented as mean (SD)
Only patients who had no missing data on exacerbations are included in the table
*Statistically significant difference between treatment and control cohorts

Supplementary Table S12. Moderate exacerbations in the subgroups of patients with and without concurrent ICS use during the first two years of treatment

	ICS use					No ICS use				
	n	Treatment cohort	n	Control cohort	P value	n	Treatment cohort	n	Control cohort	P value
Baseline	48	1.17 (1.04)	180	0.89 (1.68)	0.005*	29	1.00 (1.20)	171	0.32 (0.70)	<0.001*
Year 1	48	0.85 (1.11)	179	0.64 (1.05)	0.203	29	0.45 (0.78)	170	0.39 (0.75)	0.698
Year 2	47	0.87 (1.12)	178	0.71 (1.38)	0.196	28	0.46 (0.84)	169	0.42 (0.93)	0.664
Year 3	38	0.84 (1.20)	150	0.73 (1.13)	0.596	24	0.46 (0.78)	142	0.59 (1.37)	0.849
Year 4	24	0.50 (0.72)	103	0.85 (1.13)	0.193	18	0.56 (0.86)	105	0.51 (0.81)	0.895
Year 5	18	0.56 (0.71)	91	0.66 (1.09)	0.871	16	0.56 (1.32)	77	0.51 (1.14)	0.906
Change from baseline										
Year 1	48	-0.31 (1.17)	179	-0.26 (1.53)	0.565	29	-0.55 (1.24)	170	0.07 (0.82)	0.012*
Year 2	47	-0.28 (0.99)	178	-0.18 (1.46)	0.378	28	-0.50 (1.04)	169	0.10 (1.00)	0.003*
Year 3	38	-0.32 (1.19)	150	-0.09 (1.57)	0.094	24	-0.38 (0.92)	142	0.27 (1.33)	0.019*
Year 4	24	-0.79 (1.29)	103	-0.03 (1.42)	0.002*	18	-0.33 (1.09)	105	0.27 (0.82)	0.014*
Year 5	18	-0.72 (1.13)	91	-0.21 (1.87)	0.028*	16	-0.25 (1.00)	77	0.29 (1.27)	0.088

Data presented as mean (SD)
Only patients who had no missing data on exacerbations are included in the table
*Statistically significant difference between treatment and control cohorts

Supplementary Table S13. Severe exacerbations in all patients and in the subgroups with cough or GOLD stages 3–4 at baseline, or ICS use and no use for 24 months

	All patients				
	n	Treatment cohort	n	Control cohort	P value
Baseline	81	0.58 (0.10)	371	0.24 (0.60)	<0.001*
Year 1	81	0.36 (0.89)	368	0.18 (0.49)	0.148
Year 2	79	0.38 (0.87)	365	0.19 (0.61)	0.029*
Year 3	66	0.33 (0.59)	307	0.19 (0.53)	0.005*
Year 4	46	0.37 (0.85)	216	0.18 (0.66)	0.007*
Year 5	38	0.34 (0.58)	174	0.29 (0.82)	0.096
Change from baseline					
Year 1	81	-0.22 (1.19)	368	-0.06 (0.62)	0.060
Year 2	79	-0.20 (1.10)	365	-0.05 (0.81)	0.081
Year 3	66	-0.24 (1.04)	307	-0.04 (0.72)	0.180
Year 4	46	-0.15 (1.19)	216	-0.03 (0.82)	0.328
Year 5	38	-0.13 (0.78)	174	0.07 (0.10)	0.368

	Cough at baseline					GOLD Stage 3 or 4 at baseline				
	n	Treatment cohort	n	Control cohort	P value	n	Treatment cohort	n	Control cohort	P value
Baseline	69	0.59 (1.02)	252	0.24 (0.63)	<0.001*	51	0.73 (1.15)	187	0.31 (0.73)	0.002*
Year 1	69	0.39 (0.94)	250	0.17 (0.50)	0.084	51	0.43 (0.86)	186	0.20 (0.53)	0.099
Year 2	67	0.39 (0.90)	249	0.19 (0.66)	0.032*	50	0.40 (0.76)	184	0.25 (0.72)	0.097
Year 3	56	0.34 (0.58)	210	0.23 (0.57)	0.053	40	0.38 (0.67)	155	0.21 (0.56)	0.051
Year 4	42	0.38 (0.88)	145	0.22 (0.77)	0.040*	30	0.47 (1.01)	106	0.28 (0.87)	0.104
Year 5	34	0.38 (0.60)	112	0.37 (0.97)	0.130	22	0.27 (0.55)	89	0.40 (1.02)	0.917
Change from baseline										
Year 1	69	-0.20 (1.23)	250	-0.07 (0.65)	0.144	51	-0.29 (1.29)	186	-0.11 (0.71)	0.242
Year 2	67	-0.21 (1.15)	249	-0.06 (0.87)	0.107	50	-0.32 (1.13)	184	-0.07 (1.02)	0.151
Year 3	56	-0.23 (1.04)	210	0.01 (0.76)	0.122	40	-0.35 (1.23)	155	-0.09 (0.82)	0.225
Year 4	42	-0.17 (1.23)	145	0.01 (0.92)	0.241	30	-0.20 (1.38)	106	0.01 (1.05)	0.173
Year 5	34	-0.12 (0.81)	112	0.14 (1.14)	0.366	22	-0.36 (0.90)	89	0.12 (1.23)	0.039*

	ICS use					No ICS use				
	n	Treatment cohort	n	Control cohort	P value	n	Treatment cohort	n	Control cohort	P value
Baseline	48	0.77 (1.17)	180	0.31 (0.71)	<0.001*	29	0.24 (0.51)	171	0.19 (0.47)	0.511
Year 1	48	0.46 (1.05)	179	0.20 (0.51)	0.18	29	0.24 (0.58)	170	0.17 (0.48)	0.542
Year 2	47	0.45 (0.10)	178	0.22 (0.53)	0.223	28	0.18 (0.48)	169	0.15 (0.69)	0.28
Year 3	38	0.40 (0.64)	150	0.23 (0.62)	0.026*	24	0.21 (0.51)	142	0.12 (0.39)	0.321
Year 4	24	0.50 (1.10)	103	0.18 (0.65)	0.023*	18	0.22 (0.43)	105	0.18 (0.69)	0.242
Year 5	18	0.44 (0.62)	91	0.25 (0.69)	0.041*	16	0.19 (0.40)	77	0.34 (0.97)	0.892
Change from baseline										
Year 1	48	-0.31 (1.43)	179	-0.11 (0.68)	0.085	29	0.00 (0.66)	170	-0.02 (0.59)	0.943
Year 2	47	-0.34 (1.32)	178	-0.08 (0.85)	0.033*	28	-0.04 (0.51)	169	-0.04 (0.80)	0.727
Year 3	38	-0.40 (1.22)	150	-0.06 (0.83)	0.213	24	0.00 (0.66)	142	-0.05 (0.56)	0.982
Year 4	24	-0.17 (1.49)	103	-0.05 (0.82)	0.388	18	-0.06 (0.64)	105	-0.01 (0.86)	0.926
Year 5	18	-0.11 (0.90)	91	-0.01 (0.89)	0.869	16	-0.13 (0.72)	77	0.16 (1.14)	0.536

Data presented as mean (SD)

Only patients who had no missing data on exacerbations are included in the table

*Statistically significant difference between treatment and control cohorts

Supplementary Table S14. Exacerbations (all, moderate, severe) in patients with GOLD stage 2 at baseline

	All exacerbations				
	n	Treatment cohort	n	Control cohort	P value
Baseline	22	1.27 (1.39)	150	0.69 (1.35)	0.024*
Year 1	22	0.82 (1.44)	148	0.50 (0.97)	0.417
Year 2	21	1.19 (1.69)	148	0.55 (1.03)	0.041*
Year 3	18	0.50 (0.79)	125	0.55 (0.97)	0.977
Year 4	11	0.55 (1.04)	91	0.79 (1.11)	0.363
Year 5	11	0.91 (1.04)	68	0.60 (1.17)	0.148
Change from baseline					
Year 1	22	-0.46 (1.65)	148	-0.18 (1.52)	0.422
Year 2	21	-0.05 (1.60)	148	-0.13 (1.47)	0.887
Year 3	18	-0.50 (1.43)	125	-0.11 (1.51)	0.221
Year 4	11	-0.36 (1.69)	91	0.10 (1.47)	0.293
Year 5	11	0.36 (0.81)	68	-0.06 (1.99)	0.277

	Moderate exacerbations					Severe exacerbations				
	n	Treatment cohort	n	Control cohort	P value	n	Treatment cohort	n	Control cohort	P value
Baseline	22	0.96 (1.17)	150	0.55 (1.29)	0.037*	22	0.32 (0.65)	150	0.15 (0.42)	0.152
Year 1	22	0.55 (0.96)	148	0.36 (0.77)	0.362	22	0.27 (1.08)	148	0.14 (0.44)	0.848
Year 2	21	0.71 (0.96)	148	0.47 (0.88)	0.185	21	0.48 (1.21)	148	0.09 (0.39)	0.031*
Year 3	18	0.39 (0.70)	125	0.42 (0.76)	0.915	18	0.11 (0.32)	125	0.14 (0.43)	0.968
Year 4	11	0.36 (0.81)	91	0.70 (1.04)	0.185	11	0.18 (0.41)	91	0.09 (0.35)	0.199
Year 5	11	0.46 (0.69)	68	0.46 (1.09)	0.498	11	0.46 (0.69)	68	0.15 (0.53)	0.015*
Change from baseline										
Year 1	22	-0.41 (1.47)	148	-0.19 (1.36)	0.697	22	-0.05 (1.13)	148	0.01 (0.52)	0.331
Year 2	21	-0.19 (0.87)	148	-0.08 (1.30)	0.261	21	0.14 (1.15)	148	-0.05 (0.51)	0.632
Year 3	18	-0.28 (1.02)	125	-0.11 (1.41)	0.227	18	-0.22 (0.65)	125	0.00 (0.57)	0.159
Year 4	11	-0.27 (1.49)	91	0.15 (1.35)	0.252	11	-0.09 (0.83)	91	-0.06 (0.57)	0.801
Year 5	11	0.18 (0.75)	68	-0.07 (1.90)	0.784	11	0.18 (0.41)	68	0.02 (0.68)	0.160

Data presented as mean (SD)
Only patients who had no missing data on exacerbations are included in the table
*Statistically significant difference between treatment and control cohorts

Supplementary Table S15. All exacerbations in subgroups of patients by phenotype at baseline

	Bronchitic phenotype at baseline				
	n	Treatment cohort	n	Control cohort	P value
Baseline	64	1.70 (1.51)	189	0.94 (1.52)	<0.001*
Year 1	64	1.06 (1.38)	187	0.73 (1.12)	0.140
Year 2	62	1.16 (1.45)	186	0.81 (1.29)	0.076
Year 3	52	1.12 (1.29)	156	0.97 (1.38)	0.282
Year 4	39	0.87 (1.24)	111	0.99 (1.43)	0.703
Year 5	31	1.07 (1.18)	95	0.93 (1.54)	0.181
Change from baseline					
Year 1	64	-0.64 (1.68)	187	-0.21 (1.63)	0.057
Year 2	62	-0.52 (1.59)	186	-0.14 (1.74)	0.094
Year 3	52	-0.54 (1.46)	156	0.01 (1.64)	0.026*
Year 4	39	-0.69 (1.78)	111	-0.03 (1.68)	0.042*
Year 5	31	-0.36 (1.36)	95	-0.15 (2.24)	0.538

	Frequent exacerbator phenotype					BCO phenotype				
	n	Treatment cohort	n	Control cohort	P value	n	Treatment cohort	n	Control cohort	P value
Baseline	39	2.92 (0.90)	74	3.11 (2.14)	0.414	18	1.83 (1.69)	43	0.79 (1.36)	0.007*
Year 1	39	1.46 (1.55)	73	1.51 (1.49)	0.757	18	1.22 (1.31)	43	0.67 (1.17)	0.086
Year 2	37	1.60 (1.54)	72	1.47 (2.12)	0.239	17	1.24 (1.44)	42	0.74 (1.17)	0.194
Year 3	29	1.55 (1.43)	61	1.80 (2.07)	0.979	13	1.23 (1.30)	37	0.84 (1.21)	0.262
Year 4	20	1.20 (1.40)	38	1.68 (1.60)	0.265	11	1.46 (1.37)	27	0.90 (1.01)	0.269
Year 5	16	1.31 (1.49)	32	1.19 (1.60)	0.672	10	1.00 (1.56)	26	0.96 (1.25)	0.985
Change from baseline										
Year 1	39	-1.46 (1.76)	73	-1.60 (2.21)	0.750	18	-0.61 (1.69)	43	-0.09 (1.17)	0.407
Year 2	37	-1.35 (1.59)	72	-1.64 (1.97)	0.329	17	-0.56 (1.77)	42	0.00 (1.47)	0.167
Year 3	29	-1.38 (1.50)	61	-1.18 (2.29)	0.965	13	-0.39 (1.81)	37	0.08 (1.04)	0.469
Year 4	20	-1.75 (1.80)	38	-1.47 (1.87)	0.436	11	-0.09 (1.51)	27	0.23 (1.17)	0.496
Year 5	16	-1.44 (1.32)	32	-2.03 (2.42)	0.266	10	-0.60 (1.43)	26	0.23 (1.42)	0.121

Data presented as mean (SD)
Only patients who had no missing data on exacerbations are included in the table
*Statistically significant difference between treatment and control cohorts

Supplementary Table S16. Moderate exacerbations in subgroups of patients by phenotype at baseline

	Bronchitic phenotype at baseline				
	n	Treatment cohort	n	Control cohort	P value
Baseline	64	1.06 (1.07)	189	0.69 (1.35)	<0.001*
Year 1	64	0.66 (1.01)	187	0.54 (0.93)	0.530
Year 2	62	0.74 (1.09)	186	0.60 (1.04)	0.389
Year 3	52	0.77 (1.11)	156	0.74 (1.23)	0.559
Year 4	39	0.46 (0.76)	111	0.76 (1.11)	0.143
Year 5	31	0.65 (1.02)	95	0.62 (1.20)	0.474
Change from baseline					
Year 1	64	-0.41 (1.19)	187	-0.16 (1.43)	0.077
Year 2	62	-0.29 (1.01)	186	-0.10 (1.49)	0.163
Year 3	52	-0.27 (1.12)	156	0.02 (1.56)	0.041*
Year 4	39	-0.51 (1.25)	111	-0.03 (1.30)	0.014*
Year 5	31	-0.23 (1.06)	95	-0.21 (1.89)	0.586

	Frequent exacerbator phenotype					BCO phenotype				
	n	Treatment cohort	n	Control cohort	P value	n	Treatment cohort	n	Control cohort	P value
Baseline	39	1.87 (0.98)	74	2.35 (2.07)	0.485	18	1.06 (0.94)	43	0.61 (1.20)	0.016*
Year 1	39	0.92 (1.18)	73	1.12 (1.31)	0.392	18	0.78 (1.06)	43	0.61 (1.03)	0.508
Year 2	37	1.11 (1.22)	72	1.18 (1.92)	0.678	17	0.53 (0.62)	42	0.57 (0.91)	0.671
Year 3	29	1.03 (1.35)	61	1.41 (1.79)	0.441	13	0.77 (0.93)	37	0.60 (0.96)	0.388
Year 4	20	0.60 (0.82)	38	1.50 (1.52)	0.018*	11	0.91 (0.83)	27	0.78 (1.01)	0.488
Year 5	16	0.81 (1.33)	32	0.91 (1.42)	0.941	10	0.80 (1.55)	26	0.46 (0.76)	0.687
Change from baseline										
Year 1	39	-0.95 (1.26)	73	-1.25 (1.94)	0.413	18	-0.28 (1.07)	43	0.00 (1.09)	0.485
Year 2	37	-0.76 (1.19)	72	-1.19 (1.77)	0.229	17	-0.47 (0.94)	42	0.00 (1.47)	0.111
Year 3	29	-0.79 (1.24)	61	-0.84 (2.27)	0.968	13	-0.23 (1.36)	37	0.00 (0.91)	0.446
Year 4	20	-1.35 (1.14)	38	-0.97 (1.84)	0.322	11	-0.09 (0.94)	27	0.26 (1.16)	0.361
Year 5	16	-1.06 (1.18)	32	-1.53 (2.42)	0.626	10	-0.20 (1.14)	26	-0.15 (1.01)	0.621

Data presented as mean (SD)
Only patients who had no missing data on exacerbations are included in the table
*Statistically significant difference between treatment and control cohorts

Supplementary Table S17. Severe exacerbations in subgroups of patients by phenotype at baseline

	Bronchitic phenotype at baseline				
	n	Treatment cohort	n	Control cohort	P value
Baseline	64	0.64 (1.05)	189	0.25 (0.65)	<0.001*
Year 1	64	0.41 (0.97)	187	0.19 (0.54)	0.197
Year 2	62	0.42 (0.93)	186	0.21 (0.72)	0.053
Year 3	52	0.35 (0.59)	156	0.23 (0.58)	0.062
Year 4	39	0.41 (0.91)	111	0.23 (0.84)	0.023*
Year 5	31	0.42 (0.62)	95	0.31 (0.80)	0.051
Change from baseline					
Year 1	64	-0.23 (1.27)	187	-0.06 (0.66)	0.073
Year 2	62	-0.23 (1.19)	186	-0.04 (0.92)	0.076
Year 3	52	-0.27 (1.04)	156	-0.01 (0.79)	0.080
Year 4	39	-0.18 (1.28)	111	0.00 (1.00)	0.266
Year 5	31	-0.13 (0.85)	95	0.06 (1.03)	0.517

	Frequent exacerbator phenotype					BCO phenotype				
	n	Treatment cohort	n	Control cohort	P value	n	Treatment cohort	n	Control cohort	P value
Baseline	39	1.05 (1.23)	74	0.76 (1.04)	0.163	18	0.78 (1.26)	43	0.19 (0.50)	0.051
Year 1	39	0.54 (1.01)	73	0.38 (0.68)	0.890	18	0.44 (0.86)	43	0.09 (0.29)	0.054
Year 2	37	0.49 (1.01)	72	0.29 (0.66)	0.648	17	0.71 (1.05)	42	0.17 (0.58)	0.016*
Year 3	29	0.52 (0.63)	61	0.39 (0.74)	0.161	13	0.46 (0.66)	37	0.24 (0.60)	0.138
Year 4	20	0.60 (1.19)	38	0.18 (0.56)	0.036*	11	0.55 (0.69)	27	0.11 (0.32)	0.018*
Year 5	16	0.50 (0.73)	32	0.28 (0.63)	0.231	10	0.20 (0.42)	26	0.50 (1.14)	0.898
Change from baseline										
Year 1	39	-0.51 (1.52)	73	-0.36 (0.95)	0.423	18	-0.33 (1.19)	43	-0.09 (0.48)	0.384
Year 2	37	-0.60 (1.34)	72	-0.44 (1.22)	0.199	17	-0.12 (1.11)	42	0.00 (0.58)	0.435
Year 3	29	-0.59 (1.38)	61	-0.34 (1.18)	0.533	13	-0.15 (1.14)	37	0.08 (0.64)	0.430
Year 4	20	-0.40 (1.67)	38	-0.50 (1.13)	0.902	11	0.00 (0.78)	27	0.04 (0.34)	0.880
Year 5	16	-0.38 (0.96)	32	-0.50 (1.22)	0.951	10	-0.40 (0.97)	26	0.39 (1.13)	0.100

Data presented as mean (SD)

Only patients who had no missing data on exacerbations are included in the table

*Statistically significant difference between treatment and control cohorts
